# Prevalence of Antimicrobial Resistance and Clonal Relationship in ESBL/AmpC-Producing *Proteus mirabilis* Isolated from Meat Products and Community-Acquired Urinary Tract Infection (UTI-CA) in Southern Brazil

**DOI:** 10.3390/antibiotics12020370

**Published:** 2023-02-10

**Authors:** Matheus Silva Sanches, Luana Carvalho Silva, Caroline Rodrigues da Silva, Victor Hugo Montini, Bruno Henrique Dias de Oliva, Gustavo Henrique Migliorini Guidone, Mara Corrêa Lelles Nogueira, Maísa Fabiana Menck-Costa, Renata Katsuko Takayama Kobayashi, Eliana Carolina Vespero, Sergio Paulo Dejato Rocha

**Affiliations:** 1 Laboratory of Bacteriology, Center of Biological Sciences, Department of Microbiology, State University of Londrina, Londrina P.O. Box 10.011, Brazil; 2 Microorganism Research Center, Health Sciences Center, Department of Dermatological, Infectious and Parasitic Diseases, Medical School of São José do Rio Preto, São José do Rio Preto P.O. Box 15.090, Brazil; 3 Laboratory of Basic and Applied Bacteriology, Center of Biological Sciences, Department of Microbiology, State University of Londrina, Londrina P.O. Box 10.011, Brazil; 4 Department of Pathology, Health Sciences Center, Clinical and Toxicological Analysis, University Hospital of Londrina, State University of Londrina, Londrina P.O. Box 10.011, Brazil

**Keywords:** *bla*_CTX-M-65_, *bla*_CMY-2_, *Proteus mirabilis*, multidrug resistance, *bla*_CTX-M-2_

## Abstract

The present study aimed to evaluate the prevalence of antimicrobial resistance and clonal relationships in *Proteus mirabilis* isolated from chicken meat, beef, pork, and community-acquired urinary tract infections (UTI-CA). Chicken meat isolates showed the highest multidrug resistance (MDR), followed by those from pork and UTI-CA, whereas beef had relatively few MDR strains. All sources had strains that carried *bla*_CTX-M-65_, whereas *bla*_CTX-M-2_ and *bla*_CMY-2_ were only detected in chicken meat and UTI-CA isolates. This indicates that chicken meat should be considered an important risk factor for the spread of *P. mirabilis* carrying *ESBL* and *AmpC*. Furthermore, ESBL/AmpC producing strains were resistant to a greater number of antimicrobials and possessed more resistance genes than non-producing strains. In addition, the antimicrobial resistance genes *qnrD*, *aac(6′)-Ib-cr*, *sul1*, *sul2*, *fosA3*, *cmlA,* and *floR* were also found. Molecular typing showed a genetic similarity between chicken meat and UTI-CA isolates, including some strains with 100% similarity, indicating that chicken can be a source of *P. mirabilis* causing UTI-CA. It was concluded that meat, especially chicken meat, can be an important source of dissemination of multidrug-resistant *P. mirabilis* in the community.

## 1. Introduction

*Proteus mirabilis* is a Gram-negative bacterium belonging to the *Morganellaceae* and is commonly found in the environment, for example in soil and wastewater, in addition to being a commensal bacterium in the gut microbiota of humans and other animals [1]. It is classified as an opportunistic pathogen with several studies demonstrating its potential to cause infections. Urinary tract infections (UTIs), both nosocomial and community acquired (UTI-CA), are the most prevalent and persistent infections caused by *P. mirabilis* [2,3]. Although the prevalence may vary by region, *P. mirabilis* is among the most commonly isolated enterobacteria in UTI cases [4] and has been reported as the second most prevalent enterobacteria in a study in southern Brazil, trailing only behind *Escherichia coli* [5].

The intestinal tracts of several animals, such as broiler chickens, act as reservoirs for *P. mirabilis* [6], which may allow the transfer of these bacteria to the slaughter line, especially during evisceration of carcasses [7,8]. The isolation of *P. mirabilis* in food has been poorly documented; however, some studies have reported its presence in meat products, especially chicken [6,9,10,11], reinforcing the hypothesis of cross-contamination in the slaughterhouse and highlighting the zoonotic potential of these strains from meat products. The presence of *P. mirabilis* in meat may represent a threat to consumer health, especially when these strains harbor genes that confer resistance to clinically important antimicrobials.

The World Health Organization (WHO) declared that antimicrobial resistance is one of the greatest threats to global health [12], in which the non-rational use of these compounds in animal production accelerates the selection of resistant strains, with prospects for increase considerable in the coming years because of the intensification in the production of animal foods [13,14]. The selection and dissemination of antimicrobial-resistant bacteria is estimated to result in 10 million deaths annually by the year 2050, resulting in considerable economic loss [15]. Thus, studies aimed at monitoring antimicrobial resistance, in farm animals and their products and in human clinical practice, are of paramount importance in providing data that enables a better understanding of resistance and directing more effective interventions.

One of the major concerns are β-lactam-resistant strains that harbor genes for extended spectrum β-lactamases (ESBL) and the AmpC-type β-lactamases (AmpC). β-lactamases make treatment of infections more difficult since they inactivate several classes of antimicrobials, including penicillins, cephalosporins, and monobactams [16]. The genes that encode these β-lactamases are often found on mobile genetic elements, such as plasmids, facilitating horizontal transmission and global dissemination of these enzymes [17]. CTX-M, an ESBL variant, and CMY-2, an AmpC variant, have been highlighted as being globally widespread β-lactam-resistant strains, making them a cause for concern [16,18].

Therefore, this study aimed to evaluate the genotypic and phenotypic profile of antimicrobial resistance in *P. mirabilis* isolated from chicken, pork, beef, and UTI-CA. The focus was on ESBL- and AmpC-producing strains and their clonal relationship and association with other resistance genes, to establish if meats can be important sources of dissemination of antimicrobial resistant *P. mirabilis* in the community.

## 2. Results

### 2.1. Phenotypic Resistance to Antimicrobials

**Figure 1:**
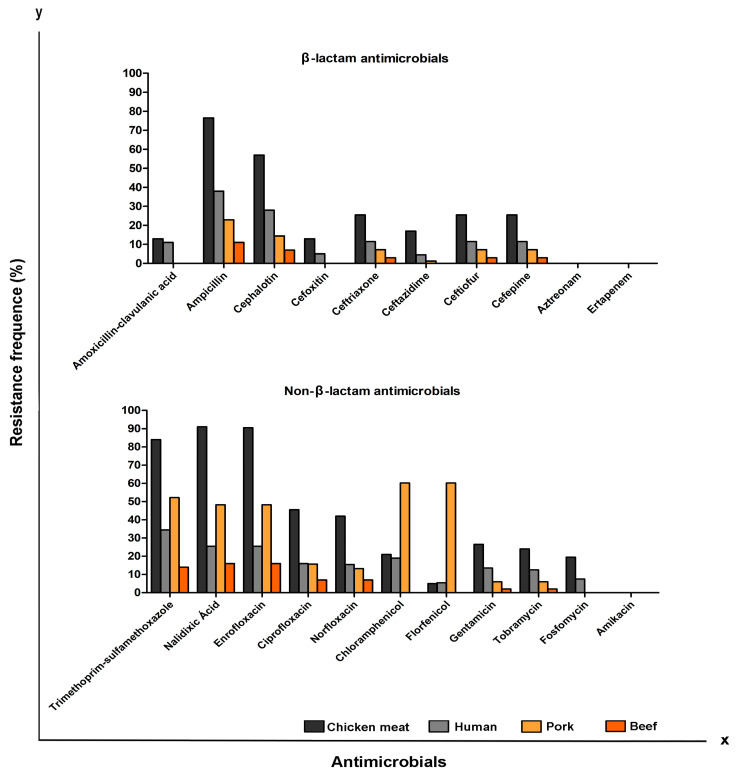
Comparison of the prevalence (%) of antimicrobial resistance in *P. mirabilis* isolated from chicken (*n* = 200), pork (*n* = 83), beef (*n* = 100), and UTI-CA (*n* = 200).

A total of 21 antimicrobials belonging to various classes were included in this study. The resistance profile of the isolates ranged from strains sensitive to all antimicrobials tested in chicken meat (*n* = 10), UTI-CA (*n* = 81), beef (*n* = 78) and pork (*n* = 28) to strains resistant to 17 antimicrobials in chicken meat and UTI-CA, 14 in swine, and 12 in beef. The resistance profile of the strains is shown in Supplementary Table S1. Chicken meat isolates exhibited higher rates of resistance to antimicrobials compared to isolates from beef, pork, and UTI-CA, except in the case of chloramphenicol and florfenicol, where pork isolates exhibited higher resistance rates (Figure 1). Statistical analysis confirmed that chicken isolates exhibited resistance to a greater number of antimicrobials than isolates from beef, pork, and UTI-CA (*p* < 0.05). Furthermore, pork isolates exhibited greater resistance to nalidixic acid, enrofloxacin, sulfamethoxazole-trimethoprim, chloramphenicol, and florfenicol than isolates from beef and UTI-CA (*p* < 0.05). Finally, UTI-CA isolates were more resistant to antimicrobials than beef isolates (*p* < 0.05).

**Figure 2:**
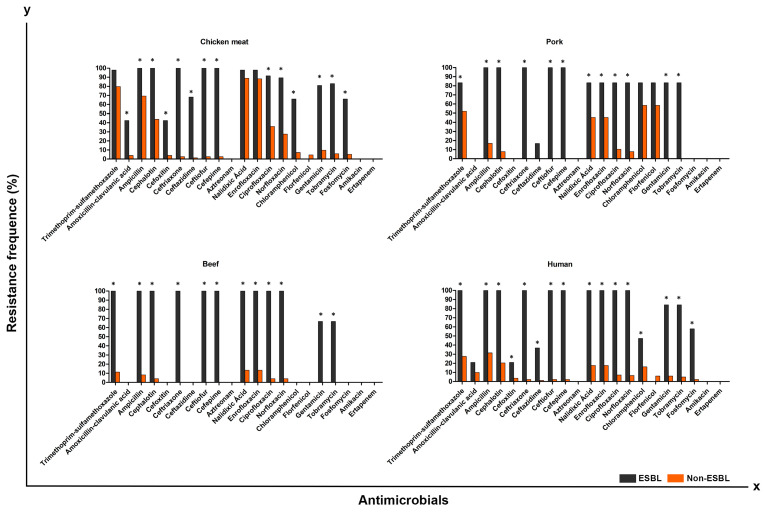
Antimicrobial resistance exhibited by ESBL-producing and non-ESBL-producing *P. mirabilis* strains isolated from chicken, pork, beef, and UTI-CA. * *p* < 0.05 by Fisher test.

**Figure 3:**
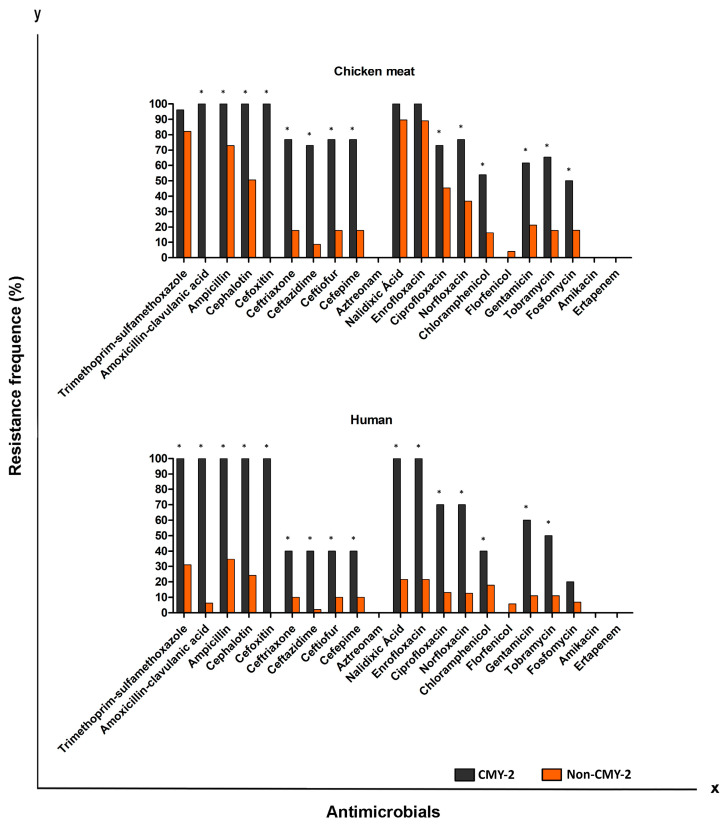
Antimicrobial resistance exhibited by AmpC-producing and non-AmpC-producing *P. mirabilis* strains isolated from chicken, pork, beef, and UTI-CA. * *p* < 0.05 by Fisher test.

Strains considered to be MDR showed a prevalence of 153 (76.5%) in chicken meat, 38 (46%) in pork, 6 (6%) in beef, and 59 (29.5%) in UTI-CA. Therefore, MDR strains tended to be more commonly found in chicken meat (OR: 9.75; CI: 5.95–13.17), followed by pork (OR: 4.13; CI: 2.57–6.64) and UTI-CA (OR: 2.8; IC: 1.82–4.31). The ESBL phenotype was confirmed in 47 chicken isolates (23.5%), 6 in pork (7.2%), 3 in beef (3%), and 19 in UTI-CA (9.5%). Regardless of the source of isolation, *P. mirabilis* carrying ESBL/AmpC were resistant to a greater number of antimicrobials (*p* < 0.05) when compared to non-producing strains (Figure 2 and Figure 3).

### 2.2. Detection of Antimicrobial Resistance Genes

All strains that expressed the ESBL phenotype were characterized by the production of CTX-M and the absence of SHV and TEM. Only the *bla*_CTX-M-2_ and *bla*_CTX-M-65_ variants (CTX-M-9 group) were detected in our study, the latter being the most prevalent variant in isolates from meat strains and UTI-CA (Table 1). No strain carried the CTX-M-2 and CTX-M-65 variants concurrently. Regarding AmpC, only *bla*_CMY-2_ was detected in chicken and UTI-CA, whereas no isolates of beef and pork harbored AmpC (Table 1). 

*P. mirabilis* isolates with *bla*_CTX-M_ (OR: 2.54; CI: 1.76–3.64) and *bla*_CMY-2_ (OR: 5.33; CI: 2.5–11.34) were more commonly found in chicken than in beef, pork, and UTI-CA. Although the more prevalence of ESBL/AmpC-producing strains in chicken meat is notable, it should be considered that chicken meat had significantly more *P. mirabilis* than pork and beef, which may have contributed to this result. More UTI-CA isolates were found to harbor *bla*_CTX-M_ (OR: 1.41; CI: 0.95–2.08) and *bla*_CMY-2_. (OR: 1.65; CI: 0.3–1.41) compared to beef and pork isolates. Of the 26 *bla*_CMY-2_-producing isolates from chicken, 20 (76.9%) harbored a variant of *bla*_CTX-M_. Of the ten *bla*_CMY-2_-producing strains in UTI-CA, four (40%) also harbored *bla*_CTX-M_. This shows that the *bla*_CMY-2_ gene is more commonly found in *bla*_CTX-M_-producing strains than in non-producing strains, both in chicken (OR: 5.48; IC: 3.06–9.81) and UTI-CA (OR: 3.58; 1.51–8.48).

In addition to the detection of *bla* genes, other genes that confer resistance to non-β-lactam antimicrobials were also found in both chicken and UTI-CA, including *fosA3*, *sul1*, *sul2*, *cmlA*, *floR*, *qnrD*, and *aac(6′)-Ib-cr*. However, some of these genes were not detected in beef or pork isolates (Table 1). The *fosA3*, *qnrD*, *sul1*, and *sul2* genes were more prevalent in chicken strains than in other sources (*p* < 0.05), whereas the *floR* gene was more prevalent in pork strains (*p* < 0.05). Pork strains also harbored more *qnrD* and *sul2* genes than beef strains and UTI-CA (*p* < 0.05). Furthermore, *bla*_CTX-M-_ and *bla*_CMY-2_-producing strains tend to have a greater number of resistance genes than the non-producing strains (Table 2). Interestingly, the *fosA3* gene was detected only in *bla*_CTX-M-65_-producing strains, showing a direct correlation (r = 0.8711923) between *bla*_CTX-M-65_ and *fosA3* in both chicken and UTI-CA (OR: >150; CI: 0.89–1.21). The *qnrA*, *qnrB*, *qnrC*, *oqxA*, *oqxB*, and *cat* genes were not detected in any isolate.

### 2.3. Molecular Typing of Isolates

**Figure 4:**
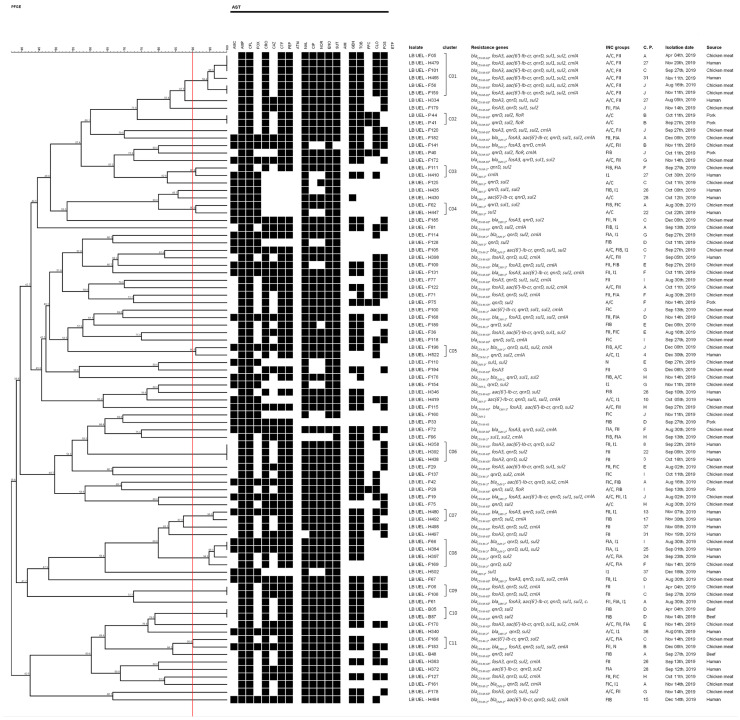
Dendrogram obtained from Not I-PFGE typing of the 87 *P. mirabilis* ESBL-AmpC producers. Dendrogram was constructed using optimization 0% and tolerance 1.5%. SXT: sulfamethoxazole-trimethoprim; AMC: amoxicillin and clavulanate; AMP: ampicillin; CEF: cephalothin; FOX: cefoxitin; CRO: ceftriaxone; CAZ: ceftazidime; CFT: ceftiofur; CPM: cefepime; ATM: aztreonam; NAL: nalidixic acid; ENO: enrofloxacin; CIP: ciprofloxacin; NOR: norfloxacin; CHL: chloramphenicol; FFC: florfenicol; GEN: gentamicin; TOB: tobramycin; FOS: fosfomycin; AMI: amikacin; ETP: ertapenem; C.P: collect point.

PFGE of chromosomal DNA digested with Not I from the 87 *bla* gene containing isolates showed 11 clusters and 76 unique pulse types, demonstrating a high heterogeneity between strains (Figure 4). Among the 11 clusters that housed genetically related strains (similarity > 90%), clusters C01, C03, C04, C05, and C08 housed isolates from chicken and UTI-CA. No clusters harbored isolates from UTI-CA and beef or pork strains. The C01 cluster was composed of four chicken strains and two UTI-CA strains, all exhibiting the same phenotypic and genotypic antimicrobial resistance profile and the same plasmid replicon. Clusters C03, C04, C05, and C08 harbored isolates from chicken and UTI-CA, showing different plasmid resistance and replicon phenotypic and genotypic profiles amongst the clusters. Cluster C02 housed two identical strains isolated from pork sourced from the same butcher shop. C06 and C07 harbored only UTI-CA strains, which had different resistance profiles and were isolated from different basic health units (BHU). Cluster C09 housed two isolates from chicken from different butcher shops but with identical characteristics. Cluster C11 housed two strains of chicken with different characteristics and C10 housed two identical isolates from beef from the same butcher shop.

## 3. Discussion

Of all the sources surveyed in our study, the highest prevalence of resistance was found in chicken isolates (Figure 1), with greater than 50% showing resistance to antimicrobials belonging to the classes of penicillins, cephalosporins, sulfonamides, and quinolones. The high prevalence of antimicrobial-resistant *P. mirabilis* in chicken is a direct consequence of the use of antimicrobials in poultry [19,20]. Furthermore, although some antimicrobials are exclusive for veterinary use, such as florfenicol, enrofloxacin, and ceftiofur, they belong to the same class of antimicrobials that are used to treat infections in humans. This can cause cross-resistance, which makes actions integrated in the One Health concept essential to minimize the impact of resistance to antimicrobials [21,22]. 

It is important to highlight the high prevalence of resistance to chloramphenicol and florfenicol observed in pork isolates relative to isolates from chicken, beef, and UTI-CA (*p* < 0.05) (Figure 1). Recently, a high prevalence of resistance to chloramphenicol in swine isolates has been reported in some countries [23,24], including Brazil [25], which may be attributed to co-selection by florfenicol, which has been used for prophylactic purposes in pig farming in Brazil [25].

Among the sources researched in our study, chicken and pork were found to be an important risk factor for the spread of antimicrobial resistant *P. mirabilis* in humans, with higher prevalence of MDR compared to isolates from UTI-CA (*p* < 0.05). In addition, among the three types of meat surveyed, beef showed the fewest MDR isolates, which may be attributed to cattle rearing being an extensive production system with little use of antimicrobials, even for therapeutic purposes [22].

Our study detected a high prevalence of MDR isolates in chicken (76.5%), which was similar to the results from other studies carried out in Southern Brazil [26,27]. In addition, our study also showed an increase in antimicrobial resistance in *P. mirabilis* strains causing UTI-CA, and a study carried out by our research group in the same region in 2017 detected only 7.1% prevalence of MDR strains [28], whereas the present study, carried out with isolates from 2019, showed 29.5% MDR prevalence. The high prevalence of MDR strains detected in meat and the emergence of MDR strains in UTI-CA is alarming, as MDR strains are associated with higher mortality rates when compared to antimicrobial- sensitive strains [29]. Our study found the presence of strains carrying ESBL in all sources surveyed, but with different prevalence, with chicken showing the highest prevalence of 47 (23.5%) ESBL-producing isolates followed by UTI-CA with 19 (9.5%), indicating that chicken represents a high risk for the spread of ESBL-producing strains (OR: 2.54; CI: 1.76–3.64). The spread of ESBL-producing strains in chicken meat imported from Brazil has already been reported in Europe and Mozambique [30,31].

Among all ESBL detected in our study, the most prevalent was *bla*_CTX-M-65_ (group CTX-M-9), which was found in all sources, and the least prevalent was *bla*_CTX-M-2_ (group CTX-M-2), found only in strains isolated from chicken and human samples (Table 1). This indicates that meat strains and UTI-CA share the same ESBL variant. In contrast to these results, although *bla*_CTX-M_ alleles tend to change in a region over time, other studies carried out with *E. coli* from broiler chickens and broiler meat in the same period in Brazil reported a higher prevalence of the CTX-M-2 group, followed by CTX-M-1 and CTX-M-8, as well as the absence of the CTX-M-9 group [26,27]. The presence of *bla*_CTX-M-65_ has already been identified in *Enterobacterales* isolated from meat and humans in several countries [32,33,34,35,36,37], whereas in Brazil, this variant has only been detected in zoo animals [38]. The *bla*_CTX-M-65_ variant has become one of the most dominant variants in farm animals and their products in China [39,40]. To our knowledge, this is the first study to detect the *bla*_CTX-M-65_ variant in *P. mirabilis* isolated from chicken, pork, beef, and UTI-CA. 

The only AmpC detected in our study was *bla*_CMY-2_, which was found to be more prevalent in chicken isolates (OR: 5.33; CI: 2.5–11.34), making chicken an important risk factor for the spread of *bla*_CMY-2_, followed by UTI-CA (OR: 1.65; CI: 0.3–1.41). Pork and beef had no isolates harboring *bla*_CMY-2_. Interestingly, our study showed a high prevalence of isolates co-producing *bla*_CTX-M_ and *bla*_CMY-2_ in chicken (76.9%) and UTI-CA (40%), indicating that chicken is an important source of dissemination of strains co-producing *ESBL* and AmpC. A previous study showed similar results on the coexistence of *ESBL* and AmpC_,_ reporting a coexistence of 64.9% in *E. coli* isolated from chicken farms in Brazil [27]. Another study showed 30% of clinical isolates of *E. coli* and *Klebsiella* spp. in Iran exhibited the coexistence of *ESBL* and AmpC [41].

Notably, the *ESBL*-producing isolates in our study exhibited greater resistance to several other antimicrobials, including cephalosporins, quinolones, aminoglycosides, amphenicols, and fosfomycin (Figure 2). Similar results of *E. coli* causing UTI-CA [42] in chickens and pigs [43] were also reported. Likewise, *bla*_CMY-2_-producing strains also exhibited more resistance to several other antimicrobials compared to non-producing strains (Figure 3). These results deserve attention, as they show that the use of antimicrobials other than β-lactams in animal production can facilitate the co-selection and increase of *ESBL*-and AmpC-producing strains. Similar to our study, another study showed that *E. coli* strains producing *bla*_CMY-2_ isolated from chickens and humans have resistance to several antimicrobials other than β-lactams [44].

In addition, our study showed that meat is an important source of dissemination of antimicrobial resistance genes not related to β-lactamases (Table 1), especially chicken, which had more isolates with the *fosA3*, *sul1*, *sul2*, *cmlA*, and *aac(6′)-Ib-cr* compared to pork, beef, and UTI-CA (*p* < 0.05). It is important to highlight the high prevalence of *sul2* and *qnrD* genes, which are responsible for the high frequency of resistance to sulfonamide and quinolones, respectively, in chicken isolates. The high prevalence of the *qnrD* gene in *P. mirabilis* has also been identified in chicken meat sold in the same region [10], showing that chicken produced in Southern Brazil can spread *P. mirabilis* with a high prevalence of this gene, which is commonly found in strains of the tribe Proteeae [45]. To our knowledge, this is the first study to report the high prevalence of the *sul2* gene and the presence of *fosA3*, *cmlA*, and *floR* genes in *P. mirabilis* isolated from chicken meat. In addition, *bla*_ESB_- and AmpC-producing strains had more genes resistant to non-β-lactam antimicrobials compared to non-producing strains (Table 2). This highlights the *qnrD* and *sul2* genes, which were more prevalent in isolates with *ESBL* and AmpC in all sources studied. This indicates that the use of other antimicrobials, such as quinolones and sulfonamides, can enable the co-selection of other resistance genes.

The fact that our study identified a higher prevalence of the *fosA3* gene in chicken (*p* < 0.05) reinforces the impact of the use of antimicrobials in poultry farming. As already evidenced by Gazal et al. [27], fosfomycin has been widely used in broiler chicken farms in the state of Paraná [27]. However, the findings regarding fosfomycin resistance in this study are very alarming, since it is one of the most used antimicrobials for the treatment of UTI-CA in countries such as Brazil, Belgium, Italy, and Russia [46]. Thus, our study proposes that fosfomycin use should be prohibited in poultry farming, since this antimicrobial has been one of the few alternatives for the treatment of infections caused by bacteria that produce β-lactamases and carbapenemases [29]. 

Recently, our research group demonstrated that several strains isolated from meat were similar to strains isolated from UTI-CA using the ERIC-PCR technique. This becomes even more aggravating when we consider the various fundamental virulence factors for the establishment of UTI in humans identified in these strains in another study by our group [11]. These results make clear the potential of *P. mirabilis* isolated from meat to cause UTI-CA. However, techniques that have better discriminatory power, as well as PFGE [47], can better contribute to the molecular typing of isolates and the identification of possible clones. As shown in Figure 4, only chicken isolates had a clonal relationship with UTI-CA isolates (clusters C01, C3, C4, C5, and C8), highlighting cluster C01, in which all six strains had the *bla*_CTX-M-65_, *fosA3*, *aac(6′)-lb-cr*, *sul1*, *sul2*, and *cmlA*, with two of these strains (one from chicken meat and one from UTI-CA) exhibiting 100% similarity. It is also important to note that C8 harbored a chicken isolate and a UTI-CA isolate that exhibited 100% similarity and carried *bla*_CTX-M-2_, *bla*_CMY-2_, *qnrD*, *sul1*, and *sul2*. Therefore, our study shows that chicken is an important source of *P. mirabilis,* carrying problematic antimicrobial resistance genes with the potential to cause UTI-CA. 

## 4. Materials and Methods

### 4.1. Bacterial Isolates

In 2021, a study carried out by our research group isolated a total of 583 *P. mirabilis* strains from chicken (*n* = 200), beef (*n* = 100), pork (*n* = 83), and urine samples from humans with UTI-CA (*n* = 200) from the same geographical area (Londrina-PR/April to December 2019). All isolates selected for this study belonged to the bacteriological collection of the Laboratory of Bacteriology at the State University of Londrina (LABAC-UEL) and have been previously recorded [11].

### 4.2. Antimicrobial Susceptibility Test

The resistance profiles of all 583 isolates were evaluated by the disk diffusion method on Mueller–Hinton agar employing antimicrobials used in treatment of human infections as recommended by the Clinical and Laboratory Standards Institute (CLSI) [48]. The antimicrobials (Oxoid™, Basingstoke, Hants, UK) were ampicillin (AMP) 10 μg, amoxicillin + clavulanate (AMC) 20/10 μg, cephalothin (CEF) 30 μg, cefoxitin (CFO) 30 μg, ceftazidime (CAZ) 30 μg, ceftriaxone (CRO) 30 μg, cefepime (CPM) 30 μg, nalidixic acid (NAL) 30 μg, norfloxacin (NOR) 10 μg, ciprofloxacin (CIP) 5 μg, sulfamethoxazole-trimethoprim (SUT) 1.25/23.75 μg, aztreonam (ATM) 30 μg, chloramphenicol (CHL) 30 μg, gentamicin (GEN) 10 μg, tobramycin (TOB) 10 μg, amikacin (AMI), fosfomycin (FOS) 200 μg, and ertapenem (ETP) 10 μg. Additionally, the veterinary antimicrobials ceftiofur (CTF) 30 μg, enrofloxacin (ENO), and florfenicol (FFC) were also used [49]. The isolates were considered multidrug-resistant (MDR) when they exhibited resistance to three or more different classes of antimicrobials [50]. Strains resistant to third-generation cephalosporins were evaluated for ESBL production using the combined disk technique following CLSI recommendations [48]. *E. coli* ATCC 25922 was used for quality control.

### 4.3. Detection of Resistance Genes

The DNA of the isolates was extracted using the Pure Link^®^ Genomic DNA Mini Kit (Invitrogen^®^, Waltham, MA, USA) and used in the polymerase chain reaction (PCR) technique to detect antimicrobial resistance genes. Isolates that exhibited phenotypic resistance to third-generation cephalosporins were subjected to detection of groups encoding CTX-M-1, 2, 8, 9, and 25; TEM; and SHV-type enzymes. Isolates suspected of producing AmpC were subjected to the detection of six specific plasmid-mediated families: ACC, CIT, DHA, EBC, FOX, and MOX. Additionally, isolates that showed resistance to other classes of antimicrobials were subjected to detection of the genes *qnrA*, *qnrB*, *qnrC qnrS*, *qnrD*, *oqxA*, *oqxB*, and *qepA* (quinolone resistance); *aac(6′)-Ib-cr* (quinolone/aminoglycosides resistance); *sul1* and *sul2* (sulfonamide resistance); and *cat*, *cmlA* and *floR* (amphenicol resistance). Information regarding the primers is shown in Supplementary Table S2. The PCR products were stained with SYBR Safe (Invitrogen^®^), subjected to electrophoresis on a 2% agarose gel, and visualized in a UV light transilluminator (Vilbert Lourmat™, Collégien, France).

### 4.4. Pulsed-Field Gel Electrophoresis (PFGE)

All *ESBL* and AmpC-producing isolates were subjected to molecular typing by pulsed-field gel electrophoresis (PFGE) using a procedure described on Pulsenet (https://www.cdc.gov/pulsenet/pathogens/pfge.html) [51] (accessed on 12 July 2022), with modifications. Briefly, bacterial suspension was prepared in sterile 0.9% NaCl, from which 100 µL was centrifuged at 16,000× *g*. The pellet was resuspended in 45 µL of TE buffer (10 mM Tris-HCl, pH 8, 1 mM EDTA) and 45 µL of CleanCut™ (Bio-Rad^TM^, Hercules, CA, USA) 2% agarose solution at 56 °C. Later, the plugs were lysed with 20 mg/mL of proteinase K (Invitrogen^TM^ Waltham, MA, USA) in TE buffer at 56 °C for 18 h. After serial washing with TE buffer, the genomic DNA in the lysis solution was digested with 50 U/pellet of Not I enzyme (Invitrogen ^TM^ Waltham, MA, USA) for 24 h at 37 °C. Electrophoresis was performed in a CHEF-DR^®^ III device (Bio-Rad^TM^, Hercules, CA, USA) under the following conditions: initial exchange time = 2.2 s, final exchange time = 54.2 s, inclined angle = 120°, and field electrical power = 6 V/cm for 24 h. The electrophoresis result was visualized after staining with 0.5 µg/mL of ethidium bromide. The dendrogram was built in BioNumerics™ (Applied Maths, Sint-Martens-Latem, BE, USA) version 7.6.3 software with 0% optimization and 1% tolerance. The genetic similarity of the isolates was determined by the Dice similarity index and the dendrogram was constructed using the unweighted pair group method with arithmetic mean (UPGMA). Isolates were considered genetically related when the similarity coefficient was ≥90%.

### 4.5. Sequencing of ESBL and bla_CMY_

The *ESBL* and AmpC products were purified with the PureLink™ PCR Purification Kit (Invitrogen^TM^ Waltham, MA, USA) and subjected to Sanger ABI-PRISM 3500 XL bidirectional sequencing (Applied Biosystems^®^, Foster City, CA, USA). β-lactamase variants were identified after evaluating sequence homology using BLAST (http://blast.ncbi.nlm.nih.gov/Blast.cgi) [52] (accessed on 12 November 2021).

### 4.6. Statistical Analysis

Multivariate logistic regression analysis and odds ratios (OR) were used to assess possible correlations between the isolation sources and the chosen variables (MDR, resistance genes). The calculation of Odds Ratio was performed using the epiDisplay package (version 3.5.0.1), where the categorical data of binomial distribution, modeled by a logistic regression, working with the entire dataset of the four sources, using the stats package (version 4.1.1), and the predictive values for OR calculation were analyzed, as well as their statistical significance via the Likelihood-ratio test (LR-test). The prevalence of antimicrobial resistance between sources and differences between ESBL- and AmpC-producing strains and non-producing strains were compared using Pearson’s Chi-square and Fisher’s test. Statistical analysis was performed using R v3.6.3 software, with a 95% confidence interval (CI). Differences were considered significant at *p* < 0.05.

## 5. Conclusions

In conclusion, *P. mirabilis* isolated from chicken, pork, and beef may exhibit resistance to multiple antimicrobials of human clinical importance. Of the three types of meat evaluated, the chicken meat was the main source of *P. mirabilis* resistant to a variety of antimicrobials, including *P. mirabilis* harboring *bla*_CTX-M-65_, *bla*_CTX-M-2_, *bla*_CMY-2_, and several other resistance genes that were also found in UTI-CA strains. Furthermore, the genetic similarity between chicken meat isolates and UTI-CA isolates indicates the circulation of strains between these sources, showing that the isolation of *P. mirabilis* from meat should not be neglected. In this sense, studies aimed at the frequent monitoring of resistance to antimicrobials in animal production and their products, as well as in humans, are of paramount importance to minimize the impact of this phenomenon and ensure the one health approach.

## Figures and Tables

**Table 1:** Prevalence of resistance genes detected in *P. mirabilis* isolated from chicken (*n* = 200), pork (*n* = 83), beef (*n* = 100), and UTI-CA (*n* = 200).

Antimicrobials	Genes	Chicken Meat	Pork	Beef	Human
β-lactams	*bla*_CTX-M-2_	14 (7.0%)	0 (0.0%)	0 (0.0%)	5 (2.5%)
	*bla*_CTX-M-65_	33 (16.5%)	6 (7.2%)	3 (3.0%)	14 (7.0%)
	*bla*_CMY-2_	26 (13.0%)	0 (0.0%)	0 (0.0%)	9 (4.5%)
Fosfomycin	*fosA3*	30 (15.0%)	0 (0.0%)	0 (0.0%)	11 (5.5%)
Sulfonamides	*sul1*	57 (28.5%)	9 (10.8%)	2 (2.0%)	19 (9.5%)
	*sul2*	124 (62.0%)	31 (37.3%)	13 (13.0%)	46 (23.0%)
Amphenicols	*cmlA*	36 (18.0%)	6 (7.2%)	0 (0.0%)	21 (10.5%)
	*floR*	6 (3.0%)	50 (60.2%)	0 (0.0%)	14 (7.0%)
Quinolones	*qnrD*	125 (62.5%)	26 (31.3%)	10 (10.0%)	34 (17.0%)
Aminoglycosides/quinolones	*aac(6′)-Ib-cr*	24 (12.0%)	0 (0.0%)	0 (0.0%)	11 (5.5%)

**Table 2:** Distribution of non-β-lactam antimicrobial resistance genes in ESBL/AmpC-producing and non-ESBL/AmpC-producing *P. mirabilis* strains isolated from chicken, beef, pork and UTI-CA.

Sources	Resistance Genes							
	ESBL/AmpC	*fosA3*	*sul1*	*sul2*	*cmlA*	*floR*	*qnrD*	*aac(6′)-Ib-cr*
**Chicken meat**	ESBL	30 (64%) *	26 (55%) *	43 (91%) *	31 (66%) *	(-)	45 (96%) *	17 (36%) *
	Non-ESBL	(-)	31 (20%)	81 (53%)	5 (3%)	6 (4%)	80 (52%)	7 (5%)
	AmpC	13 (50%) *	14 (54%) *	23 (88%) *	14 (54%) *	(-)	24 (92%) *	7 (27%) *
	Non-AmpC	17 (10%)	43 (25%)	101 (58%)	22 (13%)	6 (3%)	101 (58%)	17 (10%)
**Pork**	ESBL	(-)	(-)	5 (83%) *	1 (17%)	5 (83%)	5 (83%) *	(-)
	Non-ESBL	(-)	9 (12%)	26 (34%)	5 (6%)	45 (58 %)	21 (27%)	(-)
	AmpC	(-)	(-)	(-)	(-)	(-)	(-)	(-)
	Non-AmpC	(-)	9 (11%)	31 (37%)	6 (7%)	50 (60%) *	26 (31%)	(-)
**Beef**	ESBL	0 (0%)	(-)	3 (100%) *	(-)	(-)	3 (100%) *	(-)
	Non-ESBL	0 (0)	2 (2%)	10 (10%)	(-)	(-)	7 (7%)	(-)
	AmpC	(-)	(-)	(-)	(-)	(-)	(-)	(-)
	Non-AmpC	(-)	2 (2%)	13 (13%)	(-)	(-)	10 (10%)	(-)
**Human**	ESBL	11 (58%) *	4 (21%)	19 (100%) *	9 (47%) *	(-)	19 (100%) *	6 (32%) *
	Non-ESBL	(-)	15 (8%)	27 (15%)	12 (7%)	14 (8%)	15 (8%)	5 (3%)
	AmpC	1 (10%)	4 (40%)	8 (80%) *	4 (40%)	(-)	7 (70%) *	3 (30%)
	Non-AmpC	10 (5%)	15 (8%)	38 (20%)	17 (9%)	14 (7%)	27 (14%)	8 (4%)

*: Indicates significant difference; (-): Non-β-lactam antimicrobial resistance gene not detected.

## Data Availability

Not applicable.

## Supplementary Materials

The following supporting information can be downloaded at: https://www.mdpi.com/article/10.3390/antibiotics12020370/s1, Table S1: Phenotypic and genotypic profile of antimicrobial resistance of *P. mirabilis* isolated from meat and UTI-CA; Table S2: Sequence of primers used to detect antimicrobial resistance genes (References [53,54,55,56,57,58,59,60,61,62,63] are cited in supplementary materials).
