# Supplementary material for: Prevalence of Antimicrobial Resistance and Clonal Relationship in ESBL/AmpC-Producing Proteus mirabilis Isolated from Meat Products and Community-Acquired Urinary Tract Infection (UTI-CA) in Southern Brazil
Source: Antibiotics (Basel). 2023 Feb 10;12(2):370. doi: 10.3390/antibiotics12020370 (PMC9952622; doi:10.3390/antibiotics12020370)
Supplement: Supplementary file 1 [file antibiotics-12-00370-s001.zip › supplementary Table S1.pdf]

Supplementary Table S1 - Phenotypic and genotypic profile of antimicrobial resistance of *P. mirabilis* isolated from meat and UTI-CA

Strain	Phenotypic resistance	Genotypic resistance	ESBL/AmpC	MDR
		**Chicken meat**		
LB UEL - F01	AMP, NAL, ENO, SUT	-	-	-
LB UEL - F02	AMP, NAL, CIP, NOR, ENO, SUT, CLO	*qnrD, sul2, clmA*	-	MDR
LB UEL - F03	AMP, NAL, ENO, SUT, GEN	*qnrD, sul2*	-	MDR
LB UEL - F04	AMP, CFL, NAL, ENO, SUT	-	-	MDR
LB UEL - F05	AMP, CFL, CRO, CTF, FEP, NAL, CIP, NOR, ENO, SUT, GEN, CLO, TOB, FOS	*CTX-M-65, fosA3, aac(6')-Ib-cr, qnrD, sul1, sul2, cmlA*	ESBL	MDR
LB UEL - F06	AMP, CFL, CRO, CTF, CAZ, FEP, NAL, CIP, NOR, ENO, SUT, GEN, CLO, TOB, FOS	*CTX-M-65, fosA3, qnrD, sul2, cmlA*	ESBL	MDR
LB UEL - F07	AMP, CFL, NAL, CIP, ENO, SUT	*qnrD, sul1*	-	MDR
LB UEL - F08	SUT		-	
LB UEL - F09	AMP, NAL, ENO, SUT	*qnrD, sul1, sul2*	-	MDR
LB UEL - F10	AMP, CFL, NAL, ENO, SUT	*qnrD, sul2*	-	MDR
LB UEL - F11	AMP, CFL, NAL, ENO, SUT	-	-	MDR
LB UEL - F12	NAL, ENO	-	-	
LB UEL - F13	AMP, CFL, NAL, CIP, NOR, ENO, SUT, GEN, CLO, FFC, FOS	*qnrD, sul2, floR*	-	MDR
LB UEL - F14	AMP, CFL, NAL, CIP, NOR, ENO, SUT, GEN, TOP	*aac(6')-Ib-cr, qnrD, sul1, sul2*	-	MDR
LB UEL - F15	AMP, NAL, ENO, SUT	-	-	MDR
LB UEL - F16	AMP, CFL, NAL, ENO, SUT	*qnrD, sul2*	-	MDR
LB UEL - F17	AMP, CFL, NAL, CIP, NOR, ENO, GEN	*aac(6')-Ib-cr, qnrD*	-	MDR
LB UEL - F18	AMP, CFL, NAL, CIP, NOR, ENO, SUT	*qnrD, sul2*	-	MDR
LB UEL - F19	AMC, AMP, CFL, CFO, CRO, CAZ, FEP, NAL, CIP, NOR, ENO, SUT, GEN, CLO, TOB, CTF, FOS	*CTX-M-65, fosA3, CMY-2, aac(6')-Ib-cr, qnrD, sul1, sul2, cmlA*	ESBL/AmpC	MDR
LB UEL - F20	NAL, ENO, SUT	-	-	-
LB UEL - F21	NAL, ENO, SUT	-	-	-
LB UEL - F22	AMP, CFL, NAL, ENO, SUT	*sul2*	-	MDR
LB UEL - F23	NAL, ENO, SUT	*sul2*	-	
LB UEL - F24	AMP, CFL, NAL, CIP, NOR, ENO, SUT	*qnrD, sul1*	-	MDR
LB UEL - F25	AMP, CFL, NAL, CIP, ENO, SUT	*qnrD, sul1, sul2*	-	MDR
LB UEL - F26	AMP, CFL, SUT	-	-	MDR
LB UEL - F27	AMP, NAL, CIP, NOR, ENO, SUT	*qnrD, sul2*	-	MDR

LB UEL - F28	AMP, CFL, CRO, CTF, CAZ, FEP, NAL CIP, NOR, ENO, SUT	*qnrD, sul2, MDR*	-	MDR
LB UEL - F29	AMP, CFL, CRO, CAZ, FEP, NAL, CIP, NOR, ENO, SUT, GEN, TOB, CTF, FOS	*CTX-M-65, fosA3, aac(6')-Ib-cr, qnrD, sul1, sul2*	ESBL	MDR
LB UEL - F30	AMP, CFL, NAL, ENO, SUT	*qnrD, sul2*	-	MDR
LB UEL - F31	AMP, CFL, NAL, CIP, NOR, ENO, SUT	*qnrD, sul1*	-	MDR
LB UEL - F32	AMP, NAL, ENO, SUT	*sul2*	-	MDR
LB UEL - F33	NAL, ENO, SUT	*qnrD, sul2*	-	
LB UEL - F34	NAL, CIP, NOR, ENO, SUT, GEN, TOB	*aac(6')-Ib-cr, qnrD, sul1, sul2*	-	MDR
LB UEL - F35	AMP, CFL, NAL, ENO, SUT, CLO	*sul2, cmlA*	-	MDR
LB UEL - F36	AMP, CFL, CRO, CAZ, FEP, NAL, CIP, NOR, ENO, SUT, GEN, TOB, CTF, FOS	*CTX-M-65, fosA3, aac(6')-Ib-cr, qnrD, sul1, sul2*	ESBL	MDR
LB UEL - F37	AMP, CFL, NAL, ENO, SUT		-	MDR
LB UEL - F38	AMP, NAL, ENO, SUT	*qnrD, sul1*	-	MDR
LB UEL - F39	AMP, CFL, NAL, CIP, NOR, ENO, SUT	*qnrD, sul2*	-	MDR
LB UEL - F40	AMP, NAL, ENO, SUT	*sul2*	-	MDR
LB UEL - F41	NAL, CIP, NOR, ENO, SUT	*qnrD, sul2*	-	-
LB UEL - F42	AMC, AMP, CFL, CFO, CRO, CTF, CAZ, FEP, NAL, CIP, NOR, ENO, SUT, GEN, CLO, TOB	*CTX-M-2, CMY-2, aac(6')-Ib-cr, qnrD, sul2, cmlA*	ESBL/AmpC	MDR
LB UEL - F43	AMP, CFL, CRO, FEP, NAL, CIP, NOR, ENO, SUT, CTF	*qnrD, sul1, sul2*	-	MDR
LB UEL - F44	AMP, CFL, NAL, CIP, NOR, ENO, SUT, FFC	*qnrD, sul1, sul2*	-	MDR
LB UEL - F45	NAL, ENO, SUT, GEN, CLO, TOB, FOS	*sul2, cmlA*	-	MDR
LB UEL - F46	AMP, NAL, ENO, SUT	*qnrD, sul1, sul2*	-	-
LB UEL - F47	NAL, ENO, SUT	*qnrD*	-	-
LB UEL - F48	AMP, NAL, ENO, SUT	-	-	MDR
LB UEL - F49	AMP, CFL, NAL, CIP, NOR, ENO, SUT	*qnrD, sul2*	-	MDR
LB UEL - F50	AMP, CFL, NAL, CIP, NOR, ENO, SUT	*qnrD, sul2*	-	MDR
LB UEL - F51	AMP, CFL, NAL, CIP, NOR, ENO, SUT	*qnrD, sul2*	-	MDR
LB UEL - F52	AMP, NAL, CIP, NOR, ENO, SUT	*qnrD*	-	MDR
LB UEL - F53	AMP, CFL, SUT	-	-	MDR
LB UEL - F54	AMP, NAL, ENO, SUT	*sul2*	-	MDR
LB UEL - F55	NAL, ENO	-	-	-
LB UEL - F56	AMP, CFL, CRO, FEP, NAL, CIP, NOR, ENO, SUT, GEN, CLO, TOB, CTF, FOS	*CTX-M-65, fosA3, aac(6')-Ib-cr, qnrD, sul1, sul2, cmlA*	ESBL	MDR
LB UEL - F57	AMP, NAL, CIP, NOR, ENO, SUT, FOS	*qnrD, sul2*	-	MDR
LB UEL - F58	AMP, CFL, NAL, CIP, NOR, ENO, SUT	*qnrD, sul2*	-	MDR
LB UEL - F59	AMP, NAL, CIP, ENO, SUT, CLO, FFC	*qnrD, sul2, floR*	-	MDR
LB UEL - F60	SUT	-	-	-

LB UEL - F61	AMC, AMP, CFL, CFO, CRO, CAZ, FEP, NAL, CIP, NOR, ENO, SUT, GEN, CLO, ATM, TOB, CTF, FOS	*CTX-M-65, fosA3, CMY-2, aac(6')-Ib-cr, qnrD, sul2, cmlA*	ESBL/AmpC	MDR
LB UEL - F62	AMC, AMP, CFL, CFO, NAL, CIP, NOR, ENO, SUT	*CMY-2, qnrD, sul1, sul2*	AmpC	MDR
LB UEL - F63	AMP, NAL, ENO, SUT	*qnrD*		MDR
LB UEL - F64	AMP, CFL, NAL, ENO, SUT	*qnrD, sul2*		MDR
LB UEL - F65	AMP, SUT	-	-	-
LB UEL - F66	AMC, AMP, CFL, CFO, CRO, CAZ, FEP, NAL, CIP, NOR, ENO, SUT, GEN, ATM, TOB, CTF	*CTX-M-2, CMY-2, qnrD, sul1, sul2*	ESBL/AmpC	MDR
LB UEL - F67	AMC, AMP, CFL, CFO, CRO, CAZ, FEP, NAL, CIP, NOR, ENO, SUT, GEN, CLO, TOB, CTF, FOS	*CTX-M-65, CMY-2, fosA3, qnrD, sul1, sul2, cmlA*	ESBL/AmpC	MDR
LB UEL - F68	AMP, NAL, CIP, ENO, SUT	*qnrD, sul2*		MDR
LB UEL - F69	NAL, ENO	-	-	-
LB UEL - F70	AMP, NAL, ENO, SUT	-	-	MDR
LB UEL - F71	AMP, CFL, CRO, FEP, NAL, CIP, NOR, ENO, SUT, CLO, CTF, FOS	*CTX-M-65, fosA3, qnrD, sul2, cmlA*	ESBL	MDR
LB UEL - F72	AMC, AMP, CFL, CFO, CRO, CAZ, FEP, NAL, CIP, NOR, ENO, SUT, GEN, CLO, ATM, TOB, CTF, FOS	*CTX-M-65, CMY-2, fosA3, qnrD, sul2, cmlA*	ESBL/AmpC	MDR
LB UEL - F73	-	-	-	-
LB UEL - F74	AMP, NAL, CIP, ENO, SUT	*qnrD, sul2*	-	MDR
LB UEL - F75	AMP, CFL, CRO, CAZ, FEP, NAL, CIP, NOR, ENO, SUT, CTF, FOS	*CTX-M-65, qnrD, sul2*	ESBL	MDR
LB UEL - F76	NAL, ENO, SUT	-	-	-
LB UEL - F77	AMP, CFL, CRO, CAZ, FEP, NAL, CIP, NOR, ENO, SUT, CTF, FOS, CLO	*CTX-M-65, fosA3, qnrD, sul1, sul2, cmlA*	ESBL	MDR
LB UEL - F78	-	-	-	-
LB UEL - F79	NAL, ENO	-	-	-
LB UEL - F80	AMP, CFL, NAL, ENO, SUT	*qnrD, sul2*		MDR
LB UEL - F81	AMP, CFL, CRO, CAZ, FEP, NAL, CIP, NOR, ENO, SUT, GEN, CLO, TOB, CTF	*CTX-M-65, qnrD, sul2, cmlA*	ESBL	MDR
LB UEL - F82	AMP, CFL, NAL, CIP, ENO, SUT	*qnrD, sul1, sul2*	-	MDR
LB UEL - F83	AMP, CFL, NAL, ENO, SUT	*qnrD, sul1, sul2*	-	MDR
LB UEL - F84	AMP, NAL, ENO, SUT	*qnrD, sul2*	-	MDR
LB UEL - F85	NAL, ENO, SUT	*sul1*	-	-
LB UEL - F86	NAL, ENO, SUT	*sul1*	-	-
LB UEL - F87	NAL, CIP, NOR, ENO, SUT	*qnrD*	-	-
LB UEL - F88	AMP, CFL	-	-	-
LB UEL - F89	AMP, CFL, NAL, CIP, NOR, ENO, SUT, GEN, TOB, FOS	*aac(6')-Ib-cr, qnrD, sul1, sul2*	-	MDR
LB UEL - F90	AMP, NAL, ENO, SUT	*sul1, sul2*	-	-

LB UEL - F91	AMP, NAL, CIP, NOR, ENO, SUT, FFC, FOS	*qnrD, sul2, floR*	-	MDR
LB UEL - F92	AMP, NAL, CIP, ENO, SUT	*qnrD, sul2*	-	MDR
LB UEL - F93	NAL, ENO, SUT, CLO	*sul1, cmlA*	-	MDR
LB UEL - F94	NAL, ENO	*qnrD*	-	-
LB UEL - F95	AMP, CFL, NAL, CIP, NOR, ENO, SUT	*qnrD, sul2*	-	MDR
LB UEL - F96	AMP, CFL, CRO, CAZ, FEP, SUT, GEN, CLO, ATM, TOB, CTF	*CTX-M-2, sul1, sul2, cmlA*	ESBL	MDR
LB UEL - F97	AMP, CFL, NAL, ENO, SUT	*sul2*	-	MDR
LB UEL - F98	NAL, ENO, SUT	-	-	-
LB UEL - F99	NAL, ENO	-	-	-
LB UEL - F100	AMP, CFL, CRO, CTF, FEP, NAL, CIP, NOR, ENO, SUT, GEN, CLO, TOB	*CTX-M-2, aac(6')-Ib-cr, qnrD, sul1, sul2, cmlA*	ESBL	MDR
LB UEL - F101	AMP, CFL, CRO, CTF, FEP, NAL, CIP, NOR, ENO, SUT, GEN, CLO, TOB, FOS	*CTX-M-65, fosA3, aac(6')-Ib-cr, qnrD, sul1, sul2, cmlA*	ESBL	MDR
LB UEL - F102	AMP, CFL, NAL, ENO, SUT, GEN, TOB, FOS	*qnrD, sul2*	-	MDR
LB UEL - F103	AMP, NAL, ENO, SUT	*sul2*	-	MDR
LB UEL - F104	-	-	-	-
LB UEL - F105	AMC, AMP, CFL, CFO, CRO, CTF, CAZ, FEP, NAL, CIP, NOR, ENO, SUT	*CTX-M-2, CMY-2, aac(6')-Ib-cr, qnrD, sul1, sul2*	ESBL/AmpC	MDR
LB UEL - F106	AMP, CFL, CRO, CTF, CAZ, FEP, NAL, CIP, NOR, ENO, SUT, GEN, CLO, TOB, FOS	*CTX-M-65, fosA3, qnrD, sul2, cmlA*	ESBL	MDR
LB UEL - F107	NAL, ENO, CLO	*qnrD, cmlA*	-	-
LB UEL - F108	NAL, ENO	-	-	-
LB UEL - F109	AMC, AMP, CFL, CFO, CRO, CTF, CAZ, FEP, NAL, ENO, SUT, GEN, CLO, ATM, TOB, FOS	*CTX-M-65, fosA3, CMY-2, qnrD, sul1, cmlA*	ESBL	MDR
LB UEL - F110	AMC, AMP, CFL, CFO, NAL, ENO, SUT	*CMY-2, sul1, sul2*	AmpC	MDR
LB UEL - F111	AMP, CFL, CRO, CTF, FEP, NAL, CIP, NOR, ENO, SUT	*CTX-M-2, qnrD, sul2*	ESBL	MDR
LB UEL - F112	AMP, CFL, NAL, CIP, ENO, SUT	*qnrD, sul1, sul2*	-	MDR
LB UEL - F113	AMP, NAL, ENO, SUT, GEN, CLO, FFC	*qnrD, sul1, sul2, floR*	-	MDR
LB UEL - F114	AMC, AMP, CFL, CFO, CRO, CTF, CAZ, FEP, NAL, CIP, NOR, ENO, SUT, CLO, ATM,	*CTX-M-2, CMY-2, qnrD, sul2, cmlA*	ESBL/AmpC	MDR
LB UEL - F115	AMC, AMP, CFL, CFO, CRO, CTF, CAZ, FEP, NAL, CIP, NOR, ENO, SUT, GEN, TOB, FOS	*CTX-M-65, fosA3, CMY-2, aac(6')-Ib-cr, qnrD, sul2*	ESBL/AmpC	MDR
LB UEL - F116	-	-	-	-
LB UEL - F117	-	-	-	-
LB UEL - F118	AMP, CFL, CRO, FEP, NAL, CIP, NOR, ENO, SUT, GEN, CLO, TOB, CTF	*CTX-M-65, qnrD, sul1, cmlA*	ESBL	MDR
LB UEL - F119	AMP, CFL, NAL, CIP, NOR, ENO, SUT	*qnrD, sul1, sul2*	-	MDR

LB UEL - F120	AMP, CFL, CRO, FEP, NAL, CIP, NOR, ENO, SUT, GEN, CLO, TOB, CTF, FOS	*CTX-M-65, fosA3, qnrD, sul1, sul2, cmlA*	ESBL	MDR
LB UEL - F121	NAL, ENO	*qnrD*	-	-
LB UEL - F122	AMP, CFL, CRO, CAZ, FEP, NAL, CIP, NOR, ENO, SUT, GEN, CLO, TOB, CTF, FOS	*CTX-M-65, fosA3, aac(6')-Ib-cr, qnrD, sul2, cmlA*	ESBL	MDR
LB UEL - F123	NAL, NOR, ENO	*qnrD*	-	-
LB UEL - F124	AMP, NAL, ENO, SUT	-	-	MDR
LB UEL - F125	AMC, AMP, CFL, CFO, NAL, NOR, ENO, SUT	*CMY-2, qnrD, sul2*	AmpC	MDR
LB UEL - F126	NAL, CIP, ENO	*qnrD*	-	-
LB UEL - F127	AMP, CFL, CRO, CAZ, FEP, NAL, CIP, NOR, ENO, SUT, GEN, CLO, TOB, CTF, FOS	*CTX-M-65, fosA3, qnrD, sul2, cmlA*	ESBL	MDR
LB UEL - F128	AMC, AMP, CFL, CFO, NAL, ENO, SUT	*CMY-2, qnrD, sul2*	AmpC	MDR
LB UEL - F129	NAL, ENO	-	-	-
LB UEL - F130	AMP, CFL, NAL, CIP, NOR, ENO, SUT, GEN, TOB	*aac(6')-Ib-cr, qnrD, sul2*		MDR
LB UEL - F131	AMC, AMP, CFL, CFO, CRO, CAZ, FEP, NAL, CIP, NOR, ENO, SUT, GEN, CLO, ATM, TOB, CTF, FOS	*CTX-M-65, fosA3, CMY-2, aac(6')-Ib-cr, qnrD, sul2, cmlA*	ESBL/AmpC	MDR
LB UEL - F132	AMP, CFL	-	-	-
LB UEL - F133	NAL, CIP, ENO, SUT	*qnrD, sul2, sul1*	-	-
LB UEL - F134	AMP, NAL, ENO	-	-	-
LB UEL - F135	NAL, ENO	-	-	-
LB UEL - F136	AMP, CFL, NAL, CIP, NOR, ENO, SUT	*qnrD, sul2*	-	MDR
LB UEL - F137	AMP, CFL, CRO, FEP, NAL, CIP, NOR, ENO, SUT, GEN, CLO, TOB, CTF	*CTX-M-2 , qnrD, sul2, cmlA*	ESBL	MDR
LB UEL - F138	AMP, CFL, NAL, ENO, SUT	*sul2*	-	-
LB UEL - F139	AMP, NAL, ENO, SUT	*sul2*	-	MDR
LB UEL - F140	AMP, NAL, CIP, ENO, SUT	*qnrD*	-	MDR
LB UEL - F141	AMC, AMP, CFL, CFO, CRO, CAZ, FEP, NAL, ENO, GEN, CLO, TOB, CTF, FOS	*CTX-M-65, fosA3, CMY-2 , qnrD, cmlA*	ESBL/AmpC	MDR
LB UEL - F142	AMP, CFL, NAL, CIP, NOR, ENO, SUT	*qnrD, sul2*	-	MDR
LB UEL - F143	NAL, ENO, SUT	-	-	-
LB UEL - F144	AMP, CFL, NAL, CIP, NOR, ENO, SUT	*qnrD, sul2*	-	MDR
LB UEL - F145	AMP, CFL, NAL, CIP, NOR, ENO, SUT	*qnrD, sul2*	-	MDR
LB UEL - F146	NAL, ENO, SUT	-	-	-
LB UEL - F147	AMP, CFL, NAL, CIP, NOR, ENO, SUT	*qnrD, sul2*	-	MDR
LB UEL - F148	AMP, CFL, NAL, CIP, NOR, ENO, SUT	*qnrD, sul2*	-	MDR
LB UEL - F149	AMP, CFL, NAL, CIP, ENO, SUT	*qnrD, sul2*	-	MDR
LB UEL - F150	AMP, CFL, NAL, CIP, NOR, ENO, SUT	*qnrD, sul2*	-	MDR
LB UEL - F151	AMP, CFL, NAL, ENO, SUT, TOB, GEN	*qnrD, sul2*	-	MDR
LB UEL - F152	AMP, CFL, NAL, ENO, SUT	*sul1, sul2*	-	MDR

LB UEL - F153	AMP, CFL, NAL, ENO, SUT	*sul1, sul2*	-	MDR
LB UEL - F154	AMC, AMP, CFL, CFO, NAL, ENO, SUT	*CMY-2, qnrD, sul2*	-	MDR
LB UEL - F155	AMP, CFL, CRO, CTF, FEP, NAL, CIP, NOR, ENO, SUT, GEN	*aac(6')-Ib-cr, qnrD, sul2*	-	MDR
LB UEL - F156	AMP, CFL, NAL, CIP, NOR, ENO, SUT	*qnrD, sul1, sul2*	-	MDR
LB UEL - F157	-	-	-	-
LB UEL - F158	AMP, CFL, NAL, CIP, ENO, SUT	*sul2*	-	MDR
LB UEL - F159	AMP, CFL, CRO, FEP, NAL, CIP, NOR, ENO, SUT, GEN, CLO, TOB, CTF, FOS	*CTX-M-65, fosA3, qnrD, aac(6')-Ib-cr, sul1, sul2, cmlA*	ESBL	MDR
LB UEL - F160	AMC, AMP, CFL, CFO, NAL, ENO, SUT	*CMY-2*	AmpC	MDR
LB UEL - F161	AMC, AMP, CFL, CFO, CRO, CTF, CAZ, FEP, NAL, CIP, NOR, ENO, SUT, CLO, ATM	*CTX-M-2, CMY-2, qnrD, sul2, cmlA*	ESBL	MDR
LB UEL - F162	-	-	-	-
LB UEL - F163	AMP, CFL, NAL, CIP, ENO, SUT	*qnrD, sul2*	-	MDR
LB UEL - F164	AMP, NAL, ENO, SUT	*qnrD, sul2*	-	MDR
LB UEL - F165	AMP, CFL, NAL, ENO, SUT		-	MDR
LB UEL - F166	AMP, CFL, CRO, FEP, NAL, CIP, NOR, ENO, SUT, GEN, TOB, CTF	*CTX-M-2, aac(6')-Ib-cr, qnrD, sul2*	ESBL	MDR
LB UEL - F167	NAL, ENO	-	-	-
LB UEL - F168	AMC, AMP, CFL, CFO, CRO, CAZ, FEP, NAL, CIP, NOR, ENO, SUT, GEN, CLO, ATM, TOB, CTF, FOS	*CTX-M-65, fosA3, CMY-2, qnrD, sul1, sul2, cmlA*	ESBL/AmpC	MDR
LB UEL - F169	AMP, CFL, CRO, FEP, NAL, CIP, NOR, ENO, SUT, CTF	*CTX-M-2, qnrD, sul2*	ESBL	MDR
LB UEL - F170	AMP, CFL, CRO, CAZ, FEP, NAL, CIP, NOR, ENO, SUT, GEN, CLO, ATM, TOB, CTF, FOS	*CTX-M-65, fosA3, aac(6')-Ib-cr, qnrD, sul1, sul2, cmlA*	ESBL	MDR
LB UEL - F171	NAL, ENO, SUT, GEN, TOB	*sul2*	-	MDR
LB UEL - F172	AMC, AMP, CFL, CFO, CRO, CAZ, FEP, NAL, CIP, NOR, ENO, SUT, GEN, TOB, CTF, FOS	*CTX-M-65, fosA3, CMY-2, qnrD, sul1, sul2*	ESBL/AmpC	MDR
LB UEL - F173	-	-	-	-
LB UEL - F174	-	-	-	-
LB UEL - F175	NAL, ENO	-	-	-
LB UEL - F176	AMC, AMP, CFL, CFO, CRO, FEP, NAL, CIP, NOR, ENO, SUT, TOB, CTF	*CTX-M-2, CMY-2, qnrD, sul1, sul2*	ESBL/AmpC	MDR
LB UEL - F177	AMP, CFL, NAL, ENO, SUT	*Sul2*	-	-
LB UEL - F178	AMP, CFL, CRO, FEP, NAL, CIP, NOR, ENO, SUT, GEN, TOB, CTF, FOS	*CTX-M-65, fosA3, qnrD, sul1, sul2*	ESBL	MDR
LB UEL - F179	AMP, CFL, CRO, CAZ, FEP, NAL, CIP, NOR, ENO, SUT, GEN, TOB, CTF, FOS	*CTX-M-65, fosA3, qnrD, sul1, sul2*	ESBL	MDR
LB UEL - F180	AMP, NAL, ENO, SUT	-	-	MDR
LB UEL - F181	AMP, NAL, ENO, SUT	-	-	MDR

LB UEL - F182	AMC, AMP, CFL, CFO, CRO, CAZ, FEP, NAL, CIP, NOR, ENO, SUT, GEN, CLO, TOB, CTF, FOS	*CTX-M-65, fosA3, CMY-2, aac(6')-Ib-cr, qnrD, sul1, sul2, cmlA*	ESBL/AmpC	MDR
LB UEL - F183	AMC, AMP, CFL, CFO, CRO, CAZ, FEP, NAL, CIP, NOR, ENO, SUT, GEN, CLO, ATM, TOB, CTF, FOS	*CTX-M-65, fosA3, CMY-2, qnrD, sul1, sul2, cmlA*	ESBL/AmpC	MDR
LB UEL - F184	AMP, NAL, ENO, SUT	*qnrD, sul2*		MDR
LB UEL - F185	AMC, AMP, CFL, CFO, CRO, CAZ, FEP, NAL, CIP, NOR, ENO, SUT, GEN, ATM, TOB, CTF, FOS	*CTX-M-65, fosA3, CMY-2, qnrD, sul2*	ESBL/AmpC	MDR
LB UEL - F186	AMP, NAL, ENO, SUT, GEN, CLO	*qnrD, sul1*	-	MDR
LB UEL - F187	-	-	-	
LB UEL - F188	AMP, CFL, NAL, CIP, NOR, ENO, SUT, CLO, FFC	*qnrD, sul2, floR*	-	MDR
LB UEL - F189	AMP, CFL, CRO, FEP, NAL, CIP, ENO, SUT, GEN, TOB, CTF	*CTX-M-2, qnrD, sul2*	ESBL	MDR
LB UEL - F190	AMP, NAL, SUT	-	-	MDR
LB UEL - F191	AMP, NAL, ENO	-	-	-
LB UEL - F192	NAL, ENO	-	-	-
LB UEL - F193	AMP, NAL, CIP, NOR, ENO, SUT	*qnrD, sul1*		MDR
LB UEL - F194	AMP, CFL, CRO, CAZ, FEP, NAL, ENO, SUT, GEN, ATM, TOB, CTF, FOS	*CTX-M-65, fosA3*	ESBL	MDR
LB UEL - F195	NAL, CIP, ENO, SUT	-	-	-
LB UEL - F196	AMP, CFL, NAL, ENO, SUT	-	-	MDR
LB UEL - F197	AMP, CFL, CRO, CAZ, FEP, NAL, CIP, NOR, ENO, SUT, GEN, FFC, TOB, CTF, FOS	*aac(6')-Ib-cr, qnrD, sul1, sul2, floR*	-	MDR
LB UEL - F198	AMC, AMP, CFL, CFO, CRO, CAZ, FEP, NAL, CIP, NOR, ENO, SUT, GEN, CLO, ATM, TOB, CTF	*CTX-M-2, CMY-2, qnrD, sul1, sul2, cmlA*	ESBL/AmpC	MDR
LB UEL - F199	NAL, CIP, NOR, ENO, SUT, FOS	*qnrD, sul2*	-	MDR
LB UEL - F200	AMP, NAL, ENO, SUT, CLO	*Sul1*	-	MDR
Human				
LB UEL - H326	-	-	-	-
LB UEL - H327	AMP, CFL, NAL, ENO, SUT	*sul2*		MDR
LB UEL - H328	-	-	-	-
LB UEL - H329	AMP, SUT, CLO, FFC	*sul2, floR*		MDR
LB UEL - H330	-	-	-	-
LB UEL - H331	-	-	-	-
LB UEL - H332	AMP			
LB UEL - H333	-	-	-	-
LB UEL - H334	AMP, CFL, CRO, CAZ, FEP, NAL, CIP, NOR, ENO, SUT, GEN, ATM, TOB, CTF, FOS	*CTX-M-65, fosA3, qnrD, sul1, sul2*	ESBL	MDR
LB UEL - H335	AMP, NAL, CIP, NOR, ENO, SUT, CLO, FFC	*qnrD, sul2, cmlA, floR*	-	MDR

LB UEL - H336	-	-	-	-
LB UEL - H337	-	-	-	-
LB UEL - H338	-	-	-	-
LB UEL - H339	-	-	-	-
LB UEL - H340	AMC, AMP, CFL, CFO, CRO, CTF, CAZ, FEP, NAL, CIP, NOR, ENO, SUT, ATM	*CTX-M-2, CMY-2, qnrD, sul2*	ESBL/AmpC	MDR
LB UEL - H341	-	-	-	-
LB UEL - H342	-	-	-	-
LB UEL - H343	AMP, CFL, NAL, ENO, SUT, CLO, FFC	*qnrD, sul2, floR*	-	MDR
LB UEL - H344	-	-	-	-
LB UEL - H345	-	-	-	-
LB UEL - H346	AMP, CFL, CRO, FEP, NAL, CIP, NOR, ENO, SUT, GEN, TOB, CTF	*CTX-M-65, aac(6')-Ib-cr, qnrD, sul2*	ESBL	MDR
LB UEL - H347	AMP, CFL, SUT, GEN, TOB	*sul2*	-	MDR
LB UEL - H348	-	-	-	
LB UEL - H349	AMP, SUT, CLO, FFC	*sul2, floR*	-	MDR
LB UEL - H350	-	-	-	-
LB UEL - H351	-	-	-	-
LB UEL - H352	-	-	-	-
LB UEL - H353	-	-	-	-
LB UEL - H354	-	-	-	-
LB UEL - H355	-	-	-	-
LB UEL - H356	-	-	-	-
LB UEL - H357	NAL, CIP, NOR, ENO, SUT	*qnrD, sul1*	-	-
LB UEL - H358	AMP, CFL, CRO, FEP, NAL, CIP, NOR, ENO, SUT, GEN, TOB, CTF, FOS	*CTX-M-65, fosA3, aac(6')-Ib-cr, qnrD, sul2*	ESBL	MDR
LB UEL - H359	NAL, ENO, SUT		-	
LB UEL - H360	AMP, SUT, CLO	*sul1, sul2, cmlA*	-	MDR
LB UEL - H361	-	-	-	-
LB UEL - H362	-	-	-	-
LB UEL - H363	AMP, CFL, CRO, FEP, NAL, CIP, NOR, ENO, SUT, GEN, CLO TOB, CTF, FOS	*CTX-M-65, fosA3, qnrD, sul2, cmlA*	ESBL	MDR
LB UEL - H364	-	-	-	-
LB UEL - H365	AMP, CFL, NAL, CIP, ENO, SUT, CLO, FFC	*qnrD, sul2, floR*	-	MDR
LB UEL - H366	-	-	-	-
LB UEL - H367	-	-	-	-
LB UEL - H368	AMP, CFL	-	-	-
LB UEL - H369	-	-	-	-
LB UEL - H370	SUT	-	-	-

LB UEL - H371	-	-	-	-
LB UEL - H372	AMP, CFL, CRO, CAZ, FEP, NAL, CIP, NOR, ENO, SUT, GEN, ATM, TOB, CTF	*CTX-M-65, aac(6')-Ib-cr, qnrD, sul2*	ESBL	MDR
LB UEL - H373	AMP, CFL, NAL, ENO, SUT	*qnrD, sul2*	-	-
LB UEL - H374	-	-	-	-
LB UEL - H375	-	-	-	-
LB UEL - H376	SUT	*sul1*		
LB UEL - H377	-	-	-	-
LB UEL - H378	-	-	-	-
LB UEL - H379	AMP, NAL, ENO, SUT, CLO, FFC	*sul1, floR*	-	MDR
LB UEL - H380	AMC, AMP, CFL, NAL, ENO, SUT	-	-	MDR
LB UEL - H381	-	-	-	-
LB UEL - H382	-	-	-	-
LB UEL - H383	AMP, CFL, CRO, FEP, NAL, CIP, NOR, ENO, SUT, CLO, CTF	*sul2*	-	MDR
LB UEL - H384	AMC, AMP, CFL, CFO, CRO, CAZ, FEP, NAL, CIP, NOR, ENO, SUT, GEN, ATM, TOB, CTF	*CTX-M-2, CMY-2, , qnrD, sul1, sul2*	ESBL/AmpC	MDR
LB UEL - H385	-	-	-	-
LB UEL - H386	-	-	-	-
LB UEL - H387	-	-	-	-
LB UEL - H388	-	-	-	-
LB UEL - H389	-	-	-	-
LB UEL - H390	AMC, AMP, CFL	-	-	MDR
LB UEL - H391	-	-	-	-
LB UEL - H392	AMP, CFL, CRO, FEP, NAL, CIP, NOR, ENO, SUT, GEN, TOB, CTF, FOS	*CTX-M-65, fosA3, qnrD, sul2*	ESBL	MDR
LB UEL - H393	-	-	-	-
LB UEL - H394	-	-	-	-
LB UEL - H395	-	-	-	-
LB UEL - H396	AMC, AMP	-	-	-
LB UEL - H397	AMP, CFL, CRO, FEP, NAL, CIP, NOR, ENO, SUT, GEN, TOB, CTF	*CTX-M-2, qnrD, sul2*	ESBL	MDR
LB UEL - H398	AMP, CFL, CRO, FEP, NAL, CIP, NOR, ENO, SUT, CLO, CTF, FOS	*CTX-M-65, fosA3, qnrD, sul2, cmlA*	ESBL	MDR
LB UEL - H399	NAL, ENO	-	-	-
LB UEL - H400	-	-	-	-
LB UEL - H401	-	-	-	-
LB UEL - H402	AMP, SUT, CLO, FFC	*sul1, floR*		MDR
LB UEL - H403	AMP, CFL	-	-	-

LB UEL - H404	-	-	-	-
LB UEL - H405	AMP, NAL, CIP, NOR, ENO, SUT, GEN, CLO, TOB	*aac(6')-Ib-cr, qnrD, sul2, cmlA*	-	MDR
LB UEL - H406	AMP, CFL	-	-	
LB UEL - H407	-	-	-	-
LB UEL - H408	-	-	-	-
LB UEL - H409	-	-	-	-
LB UEL - H410	AMC, AMP, CFL, CFO, NAL, ENO, SUT, GEN, CLO, TOB	*CMY-2, cmlA*	AmpC	MDR
LB UEL - H411	-	-	-	-
LB UEL - H412	AMP, CLO, FFC	*floR*	-	-
LB UEL - H413	AMP, CFL, NAL, ENO, SUT	-	-	MDR
LB UEL - H414	AMP, CFL, SUT, GEN, CLO, TOB	*sul1, cmlA, floR*		MDR
LB UEL - H415	AMP, GEN, TOB	-	-	-
LB UEL - H416	SUT	-	-	-
LB UEL - H417	-	-	-	-
LB UEL - H418	-	-	-	-
LB UEL - H419	AMC, AMP, CFL, CFO, NAL, CIP, NOR, ENO, SUT, GEN, CLO, TOB, FOS	*CMY-2, aac(6')-Ib-cr, qnrD, sul1, sul2, cmlA*	AmpC	MDR
LB UEL - H420	AMP, SUT, CLO	*sul2, cmlA*	-	MDR
LB UEL - H421	-	-	-	-
LB UEL - H422	-	-	-	-
LB UEL - H423	-	-	-	-
LB UEL - H424	-	-	-	-
LB UEL - H425	-	-	-	-
LB UEL - H426	-	-	-	-
LB UEL - H427	AMC, AMP, CFL, GEN, CLO, TOB	-	-	MDR
LB UEL - H428	-	-	-	
LB UEL - H429	AMP, CFL, GEN, CLO, TOB	-	-	MDR
LB UEL - H430	AMC, AMP, CFL, CFO, NAL, CIP, NOR, ENO, SUT, GEN	*CMY-2, aac(6')-Ib-cr, qnrD, sul2*	AmpC	MDR
LB UEL - H431	AMC, AMP, CFL	-	-	
LB UEL - H432	AMP, NAL, ENO, SUT, CLO	*sul1, cmlA*	-	MDR
LB UEL - H433	-	-	-	-
LB UEL - H434	-	-	-	-
LB UEL - H435	AMC, AMP, CFL, CFO, NAL, CIP, NOR, ENO, SUT	*CMY-2, qnrD, sul1, sul2*	AmpC	MDR
LB UEL - H436	AMP, CFL, SUT, CLO, FFC	*sul1, floR*		MDR
LB UEL - H437	-	-	-	-
LB UEL - H438	AMP, CFL, CRO, FEP, NAL, CIP, NOR, ENO, SUT, GEN, TOB, CTF, FOS	*CTX-M-65, fosA3, qnrD, sul2*	ESBL	MDR

LB UEL - H439	-	-	-	-
LB UEL - H440	-	-	-	-
LB UEL - H441	SUT	-	-	-
LB UEL - H442	-	-	-	-
LB UEL - H443	-	-	-	-
LB UEL - H444	AMP, CFL	-	-	-
LB UEL - H445	-	-	-	-
LB UEL - H446	NAL, CIP, NOR, ENO, SUT	*qnrD, sul1, sul2*	-	
LB UEL - H447	AMC, AMP, CFL, CFO, NAL, ENO, SUT	*CMY-2, sul2*	-	MDR
LB UEL - H448	-	-	-	-
LB UEL - H449	AMC, AMP, CFL	-	-	-
LB UEL - H450	-	-	-	-
LB UEL - H451	NAL, ENO, SUT, CLO	-	-	MDR
LB UEL - H452	-	-	-	-
LB UEL - H453	-	-	-	-
LB UEL - H454	-	-	-	-
LB UEL - H455	AMC, AMP, CFL, CRO, FEP, SUT, CLO, CTF	*cmlA*	-	MDR
LB UEL - H456	-	-	-	-
LB UEL - H457	-	-	-	-
LB UEL - H458	-	-	-	-
LB UEL - H459	-	-	-	-
LB UEL - H460	AMP, CFL	-	-	-
LB UEL - H461	-	-	-	-
LB UEL - H462	SUT, CLO	*sul1, sul2, cmlA*	-	-
LB UEL - H463	SUT	-	-	-
LB UEL - H464	AMC, AMP, NAL, ENO, SUT, CLO	-	-	MDR
LB UEL - H465	AMP, CFL, CRO, FEP, NAL, CIP, NOR, ENO, SUT, GEN, CLO, TOB, CTF, FOS	*CTX-M-65, fosA3, aac(6')-Ib-cr, qnrD, sul1, sul2, cmlA*	ESBL	MDR
LB UEL - H466	AMP, CFL	-	-	-
LB UEL - H467	AMC, AMP	-	-	-
LB UEL - H468	-	-	-	-
LB UEL - H469	-	-	-	-
LB UEL - H470	AMP, CFL	-	-	-
LB UEL - H471	-	-	-	-
LB UEL - H472	SUT	*sul2*	-	-
LB UEL - H473	AMP, CFL, NAL, CIP, NOR, ENO, SUT, GEN, TOB	*aac(6')-Ib-cr, qnrD, sul2*	-	MDR
LB UEL - H474	-	-	-	-
LB UEL - H475	-	-	-	-
LB UEL - H476	-	-	-	-

LB UEL - H477	-	-	-	-
LB UEL - H478	AMC, AMP, CFL, NAL, ENO, SUT	*sul2*	-	-
LB UEL - H479	AMP, CFL, CRO, FEP, NAL, CIP, NOR, ENO, SUT, GEN, CLO, TOB, CTF, FOS	*CTX-M-65, fosA3, aac(6')-Ib-cr, qnrD, sul1, sul2, cmlA*	ESBL	MDR
LB UEL - H480	AMC, AMP, CFL, CFO, CRO, CAZ, FEP, NAL, CIP, NOR, ENO, SUT, GEN, CLO, ATM, TOB, CTF, FOS	*CTX-M-65, fosA3, CMY-2, qnrD, sul2, cmlA*	ESBL/AmpC	MDR
LB UEL - H481	AMC, AMP, CFL, CRO, CAZ, FEP, SUT, CLO, ATM, CTF	-	-	MDR
LB UEL - H482	NAL, ENO, SUT	*sul2*	-	-
LB UEL - H483	-	-	-	-
LB UEL - H484	-	-	-	-
LB UEL - H485	-	-	-	-
LB UEL - H486	-	-	-	-
LB UEL - H487	AMP, CFL, NAL, CIP, NOR, ENO, SUT, GEN, CLO, FFC, TOB, FOS	*aac(6')-Ib-cr, qnrD, sul2, floR*		MDR
LB UEL - H488	-	-	-	-
LB UEL - H489	AMP, SUT	*sul1, sul2*		
LB UEL - H490	-	-	-	-
LB UEL - H491	-	-	-	-
LB UEL - H492	AMP, CFL, CRO, FEP, NAL, CIP, NOR, ENO, SUT, GEN, CLO, TOB, CTF	*CTX-M-65, qnrD, sul2, cmlA*	ESBL	MDR
LB UEL - H493	AMP, CFL, CRO, CAZ, FEP, SUT, ATM, CTF	*sul2, floR*		MDR
LB UEL - H494	AMC, AMP, CFL, CFO, CRO, CAZ, FEP, NAL, CIP, NOR, ENO, SUT, GEN, CLO, ATM, TOB, CTF	*CTX-M-2, CMY-2, aac(6')-Ib-cr, qnrD, sul2, cmlA*	ESBL/AmpC	MDR
LB UEL - H495	-	-	-	-
LB UEL - H496	AMP	-	-	-
LB UEL - H497	AMP, CFL, CRO, CAZ, FEP, NAL, CIP, NOR, ENO, SUT, GEN, ATM, TOB, CTF, FOS	*CTX-M-65, fosA3, qnrD, sul2*	ESBL	MDR
LB UEL - H498	AMP, CFL, CRO, FEP, NAL, CIP, NOR, ENO, SUT, GEN, CLO, TOB, CTF, FOS	*CTX-M-65, fosA3, qnrD, sul2, cmlA*	ESBL	MDR
LB UEL - H499	AMP, CFL	-	-	-
LB UEL - H500	-	-	-	-
LB UEL - H501	-	-	-	-
LB UEL - H502	AMC, AMP, CFL, CFO, NAL, ENO, SUT	*CMY-2, sul1*	AmpC	MDR
LB UEL - H503	-	-	-	-
LB UEL - H504	-	-	-	-
LB UEL - H505	-	-	-	-
LB UEL - H506	AMP, CFL, NAL, ENO, SUT	*qnrD, sul1*	-	MDR
LB UEL - H507	-	-	-	-

LB UEL - H508	AMP, SUT	-	-	-
LB UEL - H509	-	-	-	-
LB UEL - H510	NAL, CIP, NOR, ENO, SUT, CLO, FFC, FOS	*qnrD, sul2, floR*	-	MDR
LB UEL - H511	-	-	-	-
LB UEL - H512	-	-	-	-
LB UEL - H513	-	-	-	-
LB UEL - H514	NAL, ENO, CLO	-	-	-
LB UEL - H515	AMP, CLO	-	-	-
LB UEL - H516	-	-	-	-
LB UEL - H517	-	-	-	-
LB UEL - H518	-	-	-	-
LB UEL - H519	AMP, CFL	-	-	-
LB UEL - H520	-	-	-	-
LB UEL - H521	AMC, AMP, NAL, CIP, NOR, ENO, SUT, CLO, FOS	*qnrD, sul2*	-	MDR
LB UEL - H522	AMP, CFL, CRO, FEP, NAL, CIP, NOR, ENO, SUT, CLO, ATM, CTF	*CTX-M-2, qnrD, sul2, cmlA*	ESBL	MDR
LB UEL - H523	-	-	-	-
LB UEL - H524	-	-	-	-
LB UEL - H525	NAL, ENO, SUT	-	-	-
		Pork		
LB UEL - P01	NAL, ENO, SUT, CLO, FFC	*qnrD, sul1, floR*	-	MDR
LB UEL - P02	NAL, ENO, SUT, CLO, FFC	*qnrD, sul1, floR*	-	MDR
LB UEL - P03	SUT, CLO, FFC	*floR*	-	-
LB UEL - P04	AMP, CFL, NAL, ENO, SUT, CLO, FFC	*sul2, floR*	-	MDR
LB UEL - P05	NAL, CIP, NOR, ENO, SUT, CLO, FFC	*qnrD, sul2, floR*	-	MDR
LB UEL - P06	AMP, NAL, ENO, CLO, FFC	*floR*	-	MDR
LB UEL - P07	-	-	-	-
LB UEL - P08	-	-	-	-
LB UEL - P09	-	-	-	-
LB UEL - P10	-	-	-	-
LB UEL - P11	-	-	-	-
LB UEL - P12	AMP, SUT, CLO, FFC	*sul2, floR*	-	MDR
LB UEL - P13	NAL, ENO, SUT, CLO, FFC	*qnrD, sul1, floR*	-	MDR
LB UEL - P14	AMP, CFL, NAL, CIP, NOR, ENO	*qnrD*	-	MDR
LB UEL - P15	NAL, ENO, SUT, CLO, FFC	*Sul2, floR, cmlA*	-	MDR
LB UEL - P16	NAL, ENO, SUT, CLO, FFC	*qnrD, sul2, floR*	-	MDR
LB UEL - P17	NAL, ENO, SUT, CLO, FFC	*qnrD, sul2, floR*	-	MDR
LB UEL - P18	AMP, CFL, NAL, CIP, ENO, SUT, CLO, FFC	*qnrD, sul2, floR*	-	MDR
LB UEL - P19	AMP, CFL, NAL, CIP, ENO, SUT, CLO, FFC	*qnrD, sul2, floR*	-	MDR

LB UEL - P20	-	-	-	-
LB UEL - P21	SUT, CLO, FFC	*floR*	-	-
LB UEL - P22	-	-	-	-
LB UEL - P23	SUT, CLO, FFC	*floR*	-	-
LB UEL - P24	NAL, ENO, SUT, CLO, FFC	*Sul2, floR*	-	MDR
LB UEL - P25	-	-	-	-
LB UEL - P26	-	-	-	-
LB UEL - P27	AMP, CFL, NAL, CIP, NOR, ENO, SUT, CLO, FFC	*qnrD, sul2, floR*	-	MDR
LB UEL - P28	-	-	-	
LB UEL - P29	AMP, CFL, CRO, CTF, FEP, NAL, CIP, NOR, ENO, SUT, GEN, CLO, FFC, TOB	*CTX-M-65, qnrD, sul2, floR*	ESBL	MDR
LB UEL - P30	NAL, ENO, SUT, CLO, FFC	*Sul2, floR*	-	MDR
LB UEL - P31	CLO, FCC	*floR*	-	-
LB UEL - P32	NAL, ENO, SUT	-	-	-
LB UEL - P33	AMP, CFL, CFO, CRO, CAZ, FEP, CTF	*CTX-M-65, CMY-2*	ESBL/AmpC	MDR
LB UEL - P34	NAL, ENO, SUT, CLO, FFC	*floR*		MDR
LB UEL - P35	-	-	-	-
LB UEL - P36	AMP, CFL, SUT, CLO, FFC	*Sul1, floR*	-	MDR
LB UEL - P37	NAL, CIP, NOR, ENO, SUT, CLO, FCC	*qnrD, sul2, floR, cmlA*	-	MDR
LB UEL - P38	NAL, CIP, NOR, ENO, SUT, CLO, FCC	*qnrD, sul2, floR, cmlA*	-	MDR
LB UEL - P39	NAL, ENO, SUT, CLO, FFC	*qnrD, sul2, floR*	-	MDR
LB UEL - P40	AMP, CFL, CRO, CTF, FEP, NAL, CIP, NOR, ENO, SUT, GEN, CLO, FFC, TOB	*CTX-M-65, qnrD, sul2, floR, cmlA*	ESBL	MDR
LB UEL - P41	AMP, CFL, CRO, CTF, FEP, NAL, CIP, NOR, ENO, SUT, GEN, CLO, FFC, TOB	*CTX-M-65, qnrD, sul2, floR*	ESBL	MDR
LB UEL - P42	NAL, ENO, SUT, CLO, FFC	*sul2, floR*	-	MDR
LB UEL - P43	AMP, NAL, ENO, SUT, CLO, FFC	*qnrD, sul1, floR*	-	MDR
LB UEL - P44	AMP, CFL, CRO, CTF, FEP, NAL, CIP, NOR, ENO, SUT, GEN, CLO, FFC, TOB	*CTX-M-65, qnrD, sul2, floR*	ESBL	MDR
LB UEL - P45	AMP, NAL, ENO, SUT, CLO, FFC	*sul1, floR*	-	MDR
LB UEL - P46	NAL, ENO, CLO, FFC	*qnrD, floR, cmlA*	-	-
LB UEL - P47	-	-	-	-
LB UEL - P48	AMP, SUT, CLO, FFC	*sul2, floR*	-	MDR
LB UEL - P49	AMP, SUT, CLO, FFC	*sul2, floR*	-	MDR
LB UEL - P50	-	-	-	-
LB UEL - P51	NAL, ENO, CLO, FFC	*floR*	-	-
LB UEL - P52	-		-	-
LB UEL - P53	NAL, ENO, CLO, FFC	*qnrD, floR, cmlA*	-	-
LB UEL - P54	-	-	-	-

LB UEL - P55	-	-	-	-
LB UEL - P56	-	-	-	-
LB UEL - P57	AMP, NAL, ENO, SUT, CLO, FFC	*qnrD, sul2, floR*	-	MDR
LB UEL - P58	AMP, NAL, ENO, SUT, CLO, FFC	*qnrD, sul2, floR*	-	MDR
LB UEL - P59	SUT, CLO, FFC	*sul2, floR*	-	-
LB UEL - P60	-	-	-	-
LB UEL - P61	SUT, CLO, FFC	*sul2, floR*	-	-
LB UEL - P62	SUT, CLO, FFC	*sul1, sul2, floR*	-	-
LB UEL - P63	-	-	-	-
LB UEL - P64	NAL, ENO	-	-	-
LB UEL - P65	-	-	-	-
LB UEL - P66	SUT, CLO, FFC	*floR*	-	-
LB UEL - P67	-	-	-	-
LB UEL - P68	NAL, ENO, SUT, CLO, FFC	*qnrD, sul2, floR*	-	MDR
LB UEL - P69	NAL, ENO, SUT, CLO, FFC	*qnrD, sul2, floR*	-	MDR
LB UEL - P70	-	-	-	
LB UEL - P71	NAL, CIP, NOR, ENO, SUT, CLO, FFC	*qnrD, sul1, floR*	-	MDR
LB UEL - P72	NAL, ENO	-	-	-
LB UEL - P73	NAL, ENO, CLO, FFC	*floR*	-	-
LB UEL - P74	-	-	-	-
LB UEL - P75	AMP, CFL, CRO, CTF, FEP, NAL, CIP, NOR, ENO, SUT, GEN, CLO, FFC, TOB	*CTX-M-65, qnrD, sul2, floR*	ESBL	MDR
LB UEL - P76	-	-	-	-
LB UEL - P77	-	-	-	-
LB UEL - P78	-	-	-	-
LB UEL - P79	-	-	-	-
LB UEL - P80	SUT, CLO, FFC	*sul2, floR*	-	-
LB UEL - P81	SUT, CLO, FFC	*sul2, floR*	-	-
LB UEL - P82	-	-	-	-
LB UEL - P83	NAL, ENO, SUT, CLO, FFC	*sul1, floR*	-	MDR
		Beef		
LB UEL - B01	NAL, CIP, NOR, ENO, SUT	*qnrD, sul2*	-	-
LB UEL - B02	-	-	-	-
LB UEL - B03	-	-	-	-
LB UEL - B04	-			
LB UEL - B05	AMP, CFL, CRO, CTF, FEP, NAL, CIP NOR, ENO, SUT, GEN, TOB	*qnrD, sul2, CTX-M-65*	ESBL	MDR
LB UEL - B06	-	-	-	-
LB UEL - B07	-	-	-	-

LB UEL - B08	-	-	-	-
LB UEL - B09	-	-	-	-
LB UEL - B10	AMP	-	-	-
LB UEL - B11	AMP	-	-	-
LB UEL - B12	-	-	-	-
LB UEL - B13	-	-	-	-
LB UEL - B14	-	-	-	-
LB UEL - B15	-	-	-	-
LB UEL - B16	-	-	-	-
LB UEL - B17	-	-	-	-
LB UEL - B18	-	-	-	-
LB UEL - B19	-	-	-	-
LB UEL - B20	NAL, ENO	-	-	-
LB UEL - B21	NAL, ENO	-	-	-
LB UEL - B22	-	-	-	-
LB UEL - B23	-	-	-	-
LB UEL - B24	-	-	-	-
LB UEL - B25	-	-	-	-
LB UEL - B26	-	-	-	-
LB UEL - B27	-	-	-	-
LB UEL - B28	-	-	-	-
LB UEL - B29	AMP, CFL	-	-	-
LB UEL - B30	-	-	-	-
LB UEL - B31	-	-	-	-
LB UEL - B32	-	-	-	-
LB UEL - B33	-	-	-	-
LB UEL - B34	-	-	-	-
LB UEL - B35	NAL, CIP, NOR, ENO	*qnrD, sul2*		
LB UEL - B36	-	-	-	-
LB UEL - B37	-	-	-	-
LB UEL - B38	-	-	-	-
LB UEL - B39	-	-	-	-
LB UEL - B40	-	-	-	-
LB UEL - B41	-	-	-	-
LB UEL - B42	-	-	-	-
LB UEL - B43	-	-	-	-
LB UEL - B44	-	-	-	-
LB UEL - B45	-	-	-	-
LB UEL - B46	-	-	-	-

LB UEL - B47	-	-	-	-
LB UEL - B48	AMP, CFL, CRO, FEP, CTF, NAL, CIP, NOR, ENO, SUT	*CTX-M-65, qnrD, sul2*	ESBL	MDR
LB UEL - B49	NAL, ENO, SUT	*qnrD, sul2*		
LB UEL - B50	-	-	-	-
LB UEL - B51	-	-	-	-
LB UEL - B52	-	-	-	-
LB UEL - B53	NAL, CIP, NOR, ENO, SUT	*qnrD, sul2*		
LB UEL - B54	-			
LB UEL - B55	-			
LB UEL - B56	NAL, ENO, SUT			
LB UEL - B58	-			
LB UEL - B59	AMP, CFL, NAL, ENO, SUT	*qnrD, sul1, sul2*		MDR
LB UEL - B60	-	-	-	-
LB UEL - B61	-	-	-	-
LB UEL - B62	-	-	-	-
LB UEL - B63	-	-	-	-
LB UEL - B64	-	-	-	-
LB UEL - B65	-	-	-	-
LB UEL - B66	-	-	-	-
LB UEL - B67	AMP, CFL			
LB UEL - B68	NAL, ENO, SUT	*qnrD, sul2*	-	-
LB UEL - B69	-	-	-	-
LB UEL - B70	-	-	-	-
LB UEL - B71	-	-	-	-
LB UEL - B72	-	-	-	-
LB UEL - B73	-	-	-	-
LB UEL - B74	AMP, NAL, ENO, SUT	*sul2*	-	MDR
LB UEL - B75	AMP, NAL, ENO, SUT	*sul2*	-	MDR
LB UEL - B76	-	-	-	-
LB UEL - B77	-	-	-	-
LB UEL - B78	-	-	-	-
LB UEL - B79	-	-	-	-
LB UEL - B80	-	-	-	-
LB UEL - B81	-	-	-	-
LB UEL - B82	AMP, CFL	-	-	-
LB UEL - B83	-	-	-	-
LB UEL - B84	-	-	-	-
LB UEL - B85	-	-	-	-

LB UEL - B86	-	-	-	-
LB UEL - B87	AMP, CFL, CRO, CTF, FEP, NAL, CIP, NOR, ENO, SUT, GEN, TOB	*CTX-M-65, qnrD, sul2*	ESBL	MDR
LB UEL - B88	-	-	-	-
LB UEL - B89	-	-	-	-
LB UEL - B90	-	-	-	-
LB UEL - B91	-	-	-	-
LB UEL - B92	NAL, CIP, NOR, ENO, SUT	*qnrD, sul2*	-	-
LB UEL - B93	-	-	-	-
LB UEL - B94	-	-	-	-
LB UEL - B95	-	-	-	-
LB UEL - B96	-	-	-	-
LB UEL - B97	-	-	-	-
LB UEL - B98	-	-	-	-
LB UEL - B99	-	-	-	-
LB UEL - B100	NAL, ENO, SUT	*sul1, sul2*		

SXT: sulfamethoxazole-trimethoprim; AMC: amoxicillin + clavulanate; AMP: ampicillin; CEF: cephalothin; FOX: cefoxitin; CRO: ceftriaxone; CAZ: ceftazidime; CFT: ceftiofur; CPM: cefepime; ATM: aztreonam; NAL: nalidixic acid; ENO: enrofloxacin; CIP: ciprofloxacin; NOR: norfloxacin; CHL: chloramphenicol; FFC: florfenicol; GEN: gentamicin; TOB: tobramycin; FOS: fosfomycin.
