# Supplementary material for: Prevalence of Antimicrobial Resistance and Clonal Relationship in ESBL/AmpC-Producing Proteus mirabilis Isolated from Meat Products and Community-Acquired Urinary Tract Infection (UTI-CA) in Southern Brazil
Source: Antibiotics (Basel). 2023 Feb 10;12(2):370. doi: 10.3390/antibiotics12020370 (PMC9952622; doi:10.3390/antibiotics12020370)
Supplement: Supplementary file 1 [file antibiotics-12-00370-s001.zip › Supplementary Table S2.pdf]

Supplementary table S2 - Sequence of primers used to detect antimicrobial resistance genes

Target	Genes	Primers sequences (5′➔3′)	(bp)	A.T	References
ESBL	CTX-M-1 group	(F) AAAAATCACTGCGCCAGT TC<br>(R) AGCTTATTCATCGCCACG TT	688	52 ºC	[53]
	CTX-M-2 group	(F) CGACGCTACCCCTGCTAT T<br>(R) CCAGCGTCAGATTTTTCAGG	404	52 ºC	
	CTX-M-8 group	(F) TCGCGTTAAGCGGATGATGC<br>(R) AACCCACGATGTGGGTAGC	326	52 ºC	
	CTX-M-9 group	(F) CAAAGAGAGTGCAACGGATG<br>(R) ATTGGAAAGCGTTCATCACC	561	52 ºC	
	CTX-M-25 group	(F) GCACGATGACATTCGGG<br>(R) AACCCACGATGTGGGTAGC	326	52 ºC	
	SHV	(F) AGCCGCTTGAGCAAATTAAAC<br>(R) ATCCCGCAGATAAATCACCAC	713	60 ºC	[54]
	TEM	(F) CATTTCCGTGTCGCCCTTATTC<br>(R)CGTTCATCCATAGTTGCCTGAC	800	60 ºC	
	CTX-M-2*	(F) ATGATGACTCAGAGCATTCG<br>(R) TGGGTTACGATTTTCGCCGC	865	58 ºC	[55]
	CTX-M-9*	(F) ATGGTGACAAAGAGAGTGCA<br>(R) CCCTTCGGCGATGATTCTC	863	58 ºC	
AmpC	ACC	(F) AACAGCCTCAGCAGCCGGTTA<br>(R)TTCGCCGCAATCATCCCTAGC	346	64 °C	[56]
	CIT	(F) TGG CCA GAA CTG ACA GGC AAA<br>(R) TTT CTC CTG AAC GTG GCT GGC	462	64 °C	
	DHA	(F) AAC TTT CAC AGG TGT GCT GGG T<br>(R) CCG TAC GCA TAC TGG CTT TGC	405	64 °C	

	EBC	(F) TCG GTA AAG CCG ATG TTG CGG<br>(R) CTT CCA CTG CGG CTG CCA GTT	302	64 °C	
	FOX	(F) AAC ATG GGG TAT CAG GGA GAT G<br>(R) CAA AGC GCG TAA CCG GAT TGG	190	64 °C	
	MOX	(F) GCT GCT CAA GGA GCA CAG GAT<br>(R) CAC ATT GAC ATA GGT GTG GTG C	520	64 °C	
	CMY-2*	(F) AACACACTGATTGCGTCTGAC<br>(R) CTGGGCCTCATCGTCAGTTA	1.226	60 °C	
	qnrA	(F) AGAGGATTTCTCACGCCAGG<br>(R) TGCCAGGCACAGATCTTGAC	580	55 ºC	
	qnrB	(F) GGMATHGAAATTCGCCACTG<br>(R) TTT GCYGYYCGCCAGTCGAA	264	55 ºC	[57]
	qnrS	(F) GCAAGTTCATTGAACAGGGT<br>(R) TCTAAACCGTCGAGTTCGGCG	428	55 ºC	
	qnrC	(F) GGGTTGTACATTTATTGAATC<br>(R) TCCACTTTACGAGGTTCT	447	50 ºC	[58]
**PMQR**	*qnrD*	(F) CGAGATCAATTTACGGGGAATA<br>(R) AACAAGCTGAAGCGCCTG	582	62 ºC	[59]
	qepA	(F) CTGCAGGTACTGCGTCATG<br>(R) CGTGTTGCTGGAGTTCTTC	403	60 ºC	
	oqxA	(F) GACAGCGTCGCACAGAATG<br>(R) GGAGACGAGGTTGGTATGGA	339	62 ºC	[60]
	oqxB	(F) CGAAGAAAGACCTCCCTACCC<br>(R) CGCCGCCAATGAGATACA	240	62 ºC	

**Quinolones/ Aminoglycosides**	*aac(6')-ib-cr*	(F) TTGCGATGCTCTATGAGTGGCTA<br>(R) CTCGAATGCCTGGCGTGTTT	482	57ºC	
**Sulfonamides**	*sul1*	(F) ACG AGA TTG TGC GGT TCT TC<br>(R) GGT TTC CGA GAT GGT GAT TG	347	55ºC	[61]
	sul2	(F) CCG TCT CGC TCG ACA GTT AT<br>(R) GTG TGT GCG GAT GAA GTC AG	506	55ºC	
**Amphenicols**	*cat*	(F) CCTGCCACTCATCGCAGT<br>(R) CCACCGTTGATATATCCC	623	60ºC	[62]
	cmlA	(F) TGTCATTTACGGCATACTCG<br>(R) ATCAGGCATCCCATTCCCAT	435	55ºC	
	floR	(F) CACGTTGAGCCTCTATAT<br>(R) ATGCAGAAGTAGAACGCG	868	55ºC	
**Fosfomicyn**	*fosA3*	(F) GGCATTTTATCAGCAGT<br>(R) AGACCATCCCCTTGTAG	350	54ºC	[63]

*: Indicates the primers that were used for the amplification and subsequent sequencing of *bla*$_{gene;}$ bp: base pairs; A.T: Anneling temperature.

References

53. Dallenne, C.; Da Costa, A.; Decré, D.; Favier, C.; Arlet, G. Development of a set of multiplex PCR assays for the detection of genes encoding important β-lactamases in Enterobacteriaceae. J. Antimicrob. Chemother. 2010, 65, 490–495, https://doi.org/10.1093/jac/dkp498.

54. Arlet, G.; Philippon, A. Construction by polymerase chain reaction and intragenic DNA probes for three main types of transferable Î²-lactamases (TEM, SHV, CARB). FEMS Microbiol. Lett. 1991, 82, 19–25, https://doi.org/10.1111/j.1574-6968.1991.tb04833.x.

55. Saladin, M.; Cao, V.T.B.; Lambert, T.; Donay, J.-L.; Herrmann, J.-L.; Ould-Hocine, Z.; Verdet, C.; Delisle, F.; Philippon, A.; Arlet, G. Diversity of CTX-M Î²-lactamases and their promoter regions fromEnterobacteriaceaeisolated in three Parisian hospitals. FEMS Microbiol. Lett. 2002, 209, 161–168, https://doi.org/10.1111/j.1574-6968.2002.tb11126.x.
56. Pérez-Pérez, F.J.; Hanson, N.D. Detection of Plasmid-Mediated AmpC β-Lactamase Genes in Clinical Isolates by Using Mul-tiplex PCR. J. Clin. Microbiol. 2002, 40, 2153–2162, https://doi.org/10.1128/jcm.40.6.2153-2162.2002.
57. Cattoir, V.; Poirel, L.; Rotimi, V.; Soussy, C.-J.; Nordmann, P. Multiplex PCR for detection of plasmid-mediated quinolone resistance qnr genes in ESBL-producing enterobacterial isolates. J. Antimicrob. Chemother. 2007, 60, 394–397, https://doi.org/10.1093/jac/dkm204.
58. Wang, M.; Guo, Q.; Xu, X.; Wang, X.; Ye, X.; Wu, S.; Hooper, D.C.; Wang, M. New Plasmid-Mediated Quinolone Resistance Gene, qnrC , Found in a Clinical Isolate of Proteus mirabilis. Antimicrob. Agents Chemother. 2009, 53, 1892–1897, https://doi.org/10.1128/aac.01400-08.
59. Cavaco, L.M.; Hasman, H.; Xia, S.; Aarestrup, F.M. qnrD , a Novel Gene Conferring Transferable Quinolone Resistance in Salmonella enterica Serovar Kentucky and Bovismorbificans Strains of Human Origin. Antimicrob. Agents Chemother. 2009, 53, 603–608, https://doi.org/10.1128/aac.00997-08.
60. Chen, X.; Zhang, W.; Pan, W.; Yin, J.; Pan, Z.; Gao, S.; Jiao, X. Prevalence of qnr , aac(6 ′)-Ib-cr , qepA , and oqxAB in Esche-richia coli Isolates from Humans, Animals, and the Environment. Antimicrob. Agents Chemother. 2012, 56, 3423–3427, https://doi.org/10.1128/aac.06191-11.
61. Li, Q.; Sherwood, J.; Logue, C. Characterization of antimicrobial resistant Escherichia coli isolated from processed bison car-casses. J. Appl. Microbiol. 2007, 103, 2361–2369, https://doi.org/10.1111/j.1365-2672.2007.03470.x.

62. Guerra, B.; Soto, S.M.; Argüelles, J.M.; Mendoza, M.C. Multidrug Resistance Is Mediated by Large Plasmids Carrying a Class 1 Integron in the Emergent Salmonella enterica Serotype [4,5,12:i:−]. Antimicrob. Agents Chemother. 2001, 45, 1305–1308, https://doi.org/10.1128/aac.45.4.1305-1308.2001.

63. Sato, N.; Kawamura, K.; Nakane, K.; Wachino, J.-I.; Arakawa, Y. First Detection of Fosfomycin Resistance Gene fosA3 in CTX-M-Producing Escherichia coli Isolates from Healthy Individuals in Japan. Microb. Drug Resist. 2013, 19, 477–482, https://doi.org/10.1089/mdr.2013.0061.
